# Influence of mental health medication on microbiota in the elderly population in the Valencian region

**DOI:** 10.3389/fmicb.2023.1094071

**Published:** 2023-03-16

**Authors:** Nicole Pesantes, Ana Barberá, Benjamí Pérez-Rocher, Alejandro Artacho, Sergio Luís Vargas, Andrés Moya, Susana Ruiz-Ruiz

**Affiliations:** ^1^Fundación para el Fomento de la Investigación Sanitaria y Biomédica de la Comunitat Valenciana (FISABIO), València, Spain; ^2^Consorcio de Investigación Biomédica en Red de Epidemiología y Salud Pública (CIBEResp), Madrid, Spain; ^3^Instituto de Biología Integrativa de Sistemas (I2Sysbio), CSIC-Universitat de València, València, Spain; ^4^Programa de Microbiología y Micología, Instituto de Ciencias Biomédicas, Facultad de Medicina, Universidad de Chile, Santiago, Chile

**Keywords:** aging, microbiota, gut-brain axis, mental health disorders, 16S rRNA gene sequencing, metagenomics, tryptophan

## Abstract

Spain has an aging population; 19.93% of the Spanish population is over 65. Aging is accompanied by several health issues, including mental health disorders and changes in the gut microbiota. The gut-brain axis is a bidirectional network linking the central nervous system with gastrointestinal tract functions, and therefore, the gut microbiota can influence an individual’s mental health. Furthermore, aging-related physiological changes affect the gut microbiota, with differences in taxa and their associated metabolic functions between younger and older people. Here, we took a case–control approach to study the interplay between gut microbiota and mental health of elderly people. Fecal and saliva samples from 101 healthy volunteers over 65 were collected, of which 28 (EE|MH group) reported using antidepressants or medication for anxiety or insomnia at the time of sampling. The rest of the volunteers (EE|NOMH group) were the control group. 16S rRNA gene sequencing and metagenomic sequencing were applied to determine the differences between intestinal and oral microbiota. Significant differences in genera were found, specifically eight in the gut microbiota, and five in the oral microbiota. Functional analysis of fecal samples showed differences in five orthologous genes related to tryptophan metabolism, the precursor of serotonin and melatonin, and in six categories related to serine metabolism, a precursor of tryptophan. Moreover, we found 29 metabolic pathways with significant inter-group differences, including pathways regulating longevity, the dopaminergic synapse, the serotoninergic synapse, and two amino acids.

## 1. Introduction

Spain has an aging population. In 2000, according to data from INE, the Spanish national statistics office (Instituto Nacional de Estadistica, 2021a)^1^, the population over 65 years of age was 16.53% and in 2022 that percentage will rise to 20.22%. In fact, demographic projections made by INE suggest that this trend is accelerating, and by 2,068 people, over 65 years of age could represent 29.4% of the population (García et al., 2021). The increase in elderly population over recent years, and the aging rate (i.e., ratio of people over 65 vs. those under 16) is currently 129.11% in Spain and the Comunidad Valenciana (Instituto Nacional de Estadistica, 2021b). This circumstance is a clear indicator of the improvement in the quality of life in post-industrial countries, but we cannot ignore the fact that the quantity of life alone is not a sufficient indicator of quality of life.

According to the World Health Organization, over 20% of adults aged 60 and over suffer from a mental or neurological disorder. Mental disorders are defined as “health conditions characterized by alterations in thinking, mood, or behavior (or a combination thereof) associated with distress and impaired functioning.” Mental health disorders affect mood, thinking, and behavior. These also include depression, anxiety, insomnia, eating disorders, and addictive behaviors. In the Comunidad Valenciana (Spain), there is a 24.6% risk of mental health disorders in adulthood, which can rise to 50% in people over 84 years old (Conselleria de Sanitat Universal i Salut Pública, 2020). Geriatric depression often remains undiagnosed and untreated and its symptoms are commonly attributed to normal aging; however, the lack of treatment has important consequences for both the patients’ quality of life and the primary care system (Park and Unützer, 2011).

The elderly may experience life stressors common to all people, but also other stressors that are more common in later life, like a significant ongoing loss in capacities and a decline in functional ability. For example, older adults may experience reduced mobility, chronic pain, frailty, or other health problems, for which they require some form of long-term care (Chen et al., 2020). In addition, older people are more likely to experience events such as bereavement, or a decline in socioeconomic status with retirement (Venkatapuram et al., 2017). All of these stressors can result in isolation, loneliness, or psychological distress in the elderly, for which they may require long-term care (Harman, 2006).

There is growing evidence that the gut-brain axis, a bidirectional communication network that links the emotional and cognitive centers of the brain with peripheral intestinal functions, plays a role in promoting mental health or disorders (Richards et al., 2021). It regulates, for instance, appetite and feeding, glucose and metabolite homeostasis, and gut motility (Cryan and O’Mahony, 2011). Several factors can influence the bidirectional interplay between the gut and the brain, including: (i) neurological diseases like Parkinson, autism spectrum disorder or Alzheimer; (ii) psychological disorders, including depression, anxiety and insomnia; and (iii) gastrointestinal (GI) disorders such as irritable bowel syndrome and obesity (Liang et al., 2018; Suganya and Koo, 2020; Richards et al., 2021).

The transmission of sensory information from the gut to the brain is mediated by hormonal and neural circuits (Suganya and Koo, 2020). After a stimulus such as ingestion, the passage of nutrients from the duodenum and jejunum produces chemical and mechanical stimuli that are detected by enteroendocrine cells (EECs). These cells will then secrete signaling peptides detected by sensory cells from the enteric nervous system (ENS) or the central nervous system (CNS) (Liang et al., 2018). There are intestinal microorganisms with the ability to produce metabolites, such as serotonin and Gamma-aminobutyric acid (GABA), which are active neurotransmitters in the human nervous system (Mazzoli and Pessione, 2016). These metabolites, once secreted by the microbiota, induce intestinal epithelial cells to release neural modulating molecules that signal the ENS, which, in turn, signals the brain function and therefore influences the hosts’ demeanor. GABA is the most abundant inhibitory neurotransmitter in the mammalian CNS. It is produced by microorganisms, plants, and animals and plays an important role in regulating blood pressure, sleep, cognition, and obesity, among other physiological functions. Therefore, it has been used as an antidepressant, hypotensive, insulin secretagogue, and as insomnia medication (Kalueff and Nutt, 2007).

It is also interesting to mention the functional role of essential amino acids produced by gut microbes, in particular tryptophan. The majority of tryptophan in the human body circulates in the blood attached to albumin, while only 10–20% can be found circulating freely (Gao et al., 2020). Studies have shown that changes in the gut microbiota affect the gut-brain axis by modulating the tryptophan metabolism and that metabolic products of tryptophan metabolism can interact with the gut-brain axis and the CNS. These metabolites include 5-hydroxytryptamine (5-HT or serotonin), indolic compounds, and kynurenines (KYN) (Gao et al., 2020). Only 1–2% of available ingested tryptophan goes through the 5-HT pathway. This has important implications as 5-HT is the neurotransmitter mainly responsible for regulating mood and anxiety.

Low serotonin levels in the CNS contribute to significantly increased depression and anxiety (Lindseth et al., 2015). The 5-HT pathway is involved in modulating emotions, food intake, sleep, sexual behavior, and pain management. Indeed, 8.95% of serotonin is synthesized in the GI tract by enterochromaffin cells (EC), which are the most common type of EECs, and help regulate intestine permeability, motility, secretion, epithelial development, mucosal inflammation, and the development and neurogenesis of the enteric nervous system (Liu et al., 2021). It is estimated that 95% of the produced serotonin is found in the GI tract (Richard et al., 2009).

The biosynthesis of 5-HT is entirely dependent on the enzyme tryptophan hydroxylase (TPH), which converts tryptophan into 5-hydroxytryptophan (5-HTP) (Gao et al., 2020). TPH is a rate-limiting enzyme that exists in TPH1 and TPH2. TPH1 is expressed in the EC cells in the GI tract and the pineal gland while TPH2 is mainly expressed in the myenteric plexus of the ENS and the serotonergic neurons of the brainstem (Pelosi et al., 2015). Dysregulation in TPH expression is believed to play a role in psychiatric disorders such as anxiety and GI diseases such as irritable bowel syndrome (Gao et al., 2020).

More than 90% of tryptophan is metabolized through the kynurenine pathway (KP). Indolamine 2, 3-dioxygenase (IDO), expressed in various organs such as the brain, the GI tract, and the liver, and tryptophan 2, 3-dioxygenase (TDO), mainly expressed in the liver, are the enzymes that catalyze the first step of tryptophan metabolism on KP (Gao et al., 2020). These enzymes transform tryptophan into N-formylkynurenine, which is subsequently metabolized into KYN. Of these enzymes, TDO mediates the metabolism of KP at a basal level, while IDO is activated in an immune-activated environment (Chen et al., 2021). After KYN biosynthesis, it will continue to form other KYN such as kynurenic acid (KYNA) and quinolinic acid (QUIN). These compounds can cross the Blood–Brain Barrier (BBB) and reach the CNS, where they can act as neuromodulators and exert either neuroprotective or neurotoxic effects (Gao et al., 2020).

Ruiz-Ruiz et al. (2020) identified a link between aging and the microbial pathway associated with tryptophan and indole (tryptophan degradation product) production and metabolism by the commensal microbiota. The key proteins involved in tryptophan-to-indole metabolism, tryptophanase (TnaA), and tryptophan synthase (TrpB) are more abundant and expressed at higher levels in the gut microbiota of infants, whereas they are expressed at significantly lower levels in adults and even lower levels or below the detection limit in the elderly. From the age of 11 years, the human gut microbiota may exhibit a decreased capacity to produce these metabolites, and from the age of 34 years, this capacity may drop by over 90% compared to childhood (Ruiz-Ruiz et al., 2020). Tryptophan deficiency from a certain age could be associated with a high risk of mental health disorders in adulthood.

Oral health is also influenced by aging, with an increased prevalence of periodontal disease (Clark et al., 2021). There are studies that have shown that the composition and diversity of the oral microbiota are related to the general health state and frailty in aging (Ogawa et al., 2018; Singh et al., 2019). Furthermore, there is strong evidence that elderly people who have a relatively high number of missing teeth are more likely to develop dementia and mild cognitive impairment (Batty et al., 2013). Also, it has been suggested that transition of bacteria from the oral mucosa to the gut is more frequent in the elderly than in adults (Iwauchi et al., 2019), which increases when volunteers suffer from inflammatory oral or intestinal diseases (Kitamoto et al., 2020). Another studies demonstrated the significance of the oral microbiome in the development or progression of a number of systemic disorders, including type 2 diabetes (Arimatsu et al., 2014) and colorectal cancer Komiya et al., 2019, which might suggest a possible effect of the oral microbiota over other disorders including mental health disorders.

In the present study, we carried out 16S rRNA gene and metagenomic sequencing to determine differences in the taxa, functions, and metabolic pathways of intestinal and oral microbiota in a cohort of over 65-year-olds in the Comunidad Valenciana (Spain). The study included individuals treated with medication for anxiety, depression, and/or insomnia and those who were not diagnosed with any mental health disorders.

## 2. Materials and methods

### 2.1. Study participants

A case–control study was performed. Fecal and saliva samples from 101 volunteers over 65 were collected (EE cohort). All participants were residents of the Comunidad Valenciana (Spain) and filled out a questionnaire about their diet, general health, habits, weight and height (with which the body mass index (BMI) has been calculated), employment situation, medical history, and vaccinations. Some of this information is collected in Supplementary Table 1. The EE cohort was composed of 37 males and 63 females (average age 71.29 ± 5.83), 28 of whom (27.72%) reported being treated with antidepressants, anxiety, or insomnia medication (EE|MH group). Of these, 24 were women corresponding to 85.7% of the group (23.8% of the complete EE cohort), and 4 were men corresponding to 14.3% of the group (4% of the complete EE cohort). The remaining 73 were controls (EE|NOMH group). All procedures were reviewed and approved by the Ethics Committee (Reference: 20210305/07) of Fundación para el Fomento de la Investigación Sanitaria y Biomédica de la Comunitat Valenciana (FISABIO). All the volunteers provided written informed consent before their participation.

### 2.2. Sample preparation

Fecal samples were collected from each volunteer in sterile tubes, containing 10 mL of RNAlater Solution (Ambion) to stabilize and preserve the integrity of nucleic acids prior analysis. Samples were homogenized by adding 10 mL phosphate-buffered saline (PBS) (containing, per liter, 8 g of NaCl, 0.2 g of KCl, 1.44 g of Na2HPO4, and 0.24 g of KH2PO4 [pH 7.2]) and then centrifuged to eliminate solid waste. The obtained fecal microbial suspension was aliquoted and stored at −80°C until further processing. With respect to saliva samples, 3 mL was collected from each volunteer in sterile containers, aliquoted, and stored at −80°C until further processing.

### 2.3. DNA extraction of fecal samples

A total of 500 μL of fecal suspension was pelleted and weighted and the total genomic DNA was extracted using the QIAamp DNA mini stool kit (Qiagen). The fecal suspension pellet was resuspended in 1 mL of inhibitEX Buffer from the extraction kit and then 20 μL of lysozyme (10 mg/mL) was added for cellular lysis, followed by 30 min incubation at 37°C. The lysate was subjected to mechanical treatment with 200 μL of 150–212 μm diameter Glass Beads (Sigma) and heated to 95°C for 5 min. The samples were then centrifuged and 600 μL of the supernatant was treated with 45 μL of proteinase K. The following steps were carried out according to the manufacturers’ recommendations.

### 2.4. DNA extraction of saliva samples

A total of 250 μL of saliva was pelleted at 4°C, weighted, and total genomic DNA was extracted using the QIAamp DNA mini kit (Qiagen) with a few preliminary steps. The pellet was resuspended in the leftover supernatant and incubated for 45 s in a 37°C ultrasonic cleaner (Raypa). Then, 130 μL of AL Buffer from the extraction kit was added to each sample and then 10 μL of “enzyme mix” containing 2.5 μL of lysozyme (100 mg/mL), 2.5 μL of lysostaphin (5 mg/mL), 2.5 μL of mutanolysin, and 2.5 μL of nuclease-free water was also added and incubated for one-hour at 37°C. Subsequently, 20 μL of proteinase K from the extraction kit was added to the lysate and the samples were incubated for 10 min at 56°C, followed by 10 min at 70°C, and 3 min at 95°C incubation. The lysate was then mixed with 200 μL of 100% ethanol and placed on the kit mini-column. Finally, the washing steps were performed according to the manufacturers’ recommendations.

### 2.5. 16S rRNA gene amplification, library, and sequencing

For fecal and saliva samples, V3-V4 hypervariable regions of the 16S rRNA gene were amplified by PCR using primers: 5′-TCGTCGGCAGCGTCAGATGTGTATAAGAGACAGCCTACGGGNGGCWGCAG-3′ (forward); and 5′-GTCTCGTGGGCTCGGAGATG TGTATAAGAGACAGGACTACHVGGTATCTAATCC-3′ (reverse). Amplicons were purified using NucleoMag NGS Clean-up and Size Select magnetic beads (Macherey-Nagel) and then Illumina sequencing adapters using the Nextera XT Index Kit (Illumina) were attached. Quantification of DNA was performed with a Qubit 3.0 fluorometer using the Qubit dsDNA HS assay kit (Thermo Fisher Scientific). Amplicon libraries were pooled in equimolar ratios for sequencing on a MiSeq platform of Illumina (2 × 300 bp paired-end reads) following the manufacturers’ recommendations.

### 2.6. Metagenome library and sequencing

For fecal samples, whole-genome sequencing was also performed from total DNA. Metagenome libraries were obtained with Illumina’s Nextera XT DNA Library Preparation Kit. Short fragments were eliminated using NucleoMag NGS Clean-up and Size Select magnetic beads (Macherey-Nagel) and the obtained purified libraries were sequenced in a MiSeq platform of Illumina (2 × 150 bp paired-end reads) following the manufacturers’ recommendations.

### 2.7. Bioinformatics and statistical analysis

In-house bioinformatic analysis pipelines were applied. For 16S rDNA gene analysis, we obtained the amplicon sequence variant (ASV) data with the DADA2 pipeline (Callahan et al., 2016), which removed the forward and reverse primers, filtered low-quality reads, and trimmed reads by length. Paired reads were merged to obtain the full denoised sequences, combined and abundance matrices were obtained. Chimeric ASVs as well as host (human) ASVs were removed. Finally, taxonomy was assigned to each variant by comparing them against the SILVA database (Quast et al., 2012) (naive Bayesian classifier to assign up to genus level and 97% blast matching for species level).

For metagenomic analysis, once the raw sequencing data were obtained, the sequencing adaptors were removed by Cutadapt software. Low-quality reads were eliminated using PRINSEQ, as well as short reads, and reads with a high percentage of ambiguous bases, in addition to low entropy reads. To join overlapping pairs to obtain longer sequences, the FLASH software was used. Non-overlapping forward pairs were also taken into account while non-overlapping reverse pairs were discarded. The host (human) genome and the non-coding ribosomal RNA sequences were filtered out using Bowtie2 with the SILVA database. The reads were then assembled into contigs using Megahit and mapped against the contigs using Blast. Open reading frames (ORF) were predicted using the Prodigal software and abundance tables were created. Functional annotation was performed by mapping each ORF against protein family database using the program HMMER and the KEGG Orthology database. Finally, Kaiju was used for taxonomical annotation of metagenome data. Once the functional compositional matrix was obtained, the results were grouped into functional categories and metabolic pathways.

Each matrix, including the ASV, phylum, genus, and functional compositional matrices were then analyzed. The R statistics software was applied to obtain alpha (Shannon and Chao1 indexes) and beta diversity (Canonical Correspondence Analysis (CCA), Permanova test, and Wilcoxon non-parametric test). Correlation analysis between the saliva and fecal samples was obtained using the sPCA mixomics approach for a single omic (Kim-Anh et al., 2016).

### 2.8. Robustness analysis: attenuation and buffering

Functional capacities of microbiomes are dependent on the taxonomic structure of the microbial community, because each taxon is associated to putatively different functions and abundances. The functional metagenome could be inferred considering the taxonomic composition of the microbial community. Changes in the composition and/or abundance of one or more taxa can cause changes in functional capacities. This has recently been described as taxa-function relationships (Vieira-Silva et al., 2016; Eng and Borenstein, 2018).

Two main systemic parameters can be estimated to determine the functional robustness of microbial communities: attenuation and buffering. The determination was done using the microbial community taxa-function robustness estimation pipeline developed by Eng and Borenstein (2018).^2^ To calculate changes in functional capacities or, more formally, to quantify the changes in gene composition induced by changes in taxonomical structure, the abovementioned work describes an approach to evaluate the taxa-function robustness and quantify the two abovementioned parameters. Attenuation measures how rapidly the functional shift increases as perturbation magnitude increases and buffering is defined as how large a taxonomic perturbation must be before noticeable functional shifts occur. These two parameters can be measured globally and for particular superpathways or pathways, thereby detecting the weakest points in the global microbiota metabolism when a stochastic change in the microbial community occurs, generating deviations in the functional profile.

We have implemented some modifications in the original pipeline in order to improve sensitivity and accuracy. First, we used PICRUSt2 (Douglas et al., 2020) to derive a 16S copy number table, a genomic content annotation table, and a phylogenetic tree. Second, those files were used to replace the ones provided by the original pipeline. Manipulation of data and its graphical representation, as well as and statistical tests, was done using R scripts using libraries dplyr, ggplot, and ggpubr. Attenuation and Buffering measurements, graphical representation, and statistical tests were done using R scripts and libraries dplyr (Wickham et al., 2022) and ggplot2 (Wickham, 2016).

### 2.9. Data availability statement

The curated sequences from 16S rRNA gene and metagenomes were deposited in the EBI Short Read Archive under the study accession number PRJEB56919, with accession numbers ERS13596619- ERS13596719 and ERS13596821-ERS13596921 for the16S rRNA gene from fecal and saliva samples, respectively, and ERS13596720-ERS13596820 for metagenomes.

## 3. Results

### 3.1. Clinical and biochemical characteristics

We obtained samples from a cohort of 101 volunteers over 65 years old (EE cohort) from the Comunidad Valenciana (Spain), 28 of whom were treated with medication for anxiety, depression, and/or insomnia (EE|NOMH group) and 73 not treated for any mental health disorders (EE|NOMH group). The medication of the EE|MH group included modulators of GABA receptors, modulators of serotonin availability, or sleep regulators (Table 1). Some participants combined more than one type of medication at the same time. In addition, some of them suffer common age-related diseases (hypercholesterolemia, hypertension, diabetes, and coronary diseases) and take medication for its treatment. Both groups had a similar representation of these most common diseases.

**Table 1:** Volunteers over 65 years from the Valencian Community which were treated with medication for anxiety, depression, and insomnia (EE|MH).

Identification number	Age	Gender	Group
EE13	67	Female	1
EE24	68	Female	1
EE25	68	Female	2
EE26	65	Male	2
EE27	68	Female	4
EE34	70	Female	3
EE37	75	Female	1
EE38	83	Female	1
EE40	69	Female	2
EE42	65	Female	1
EE50	65	Female	2
EE54	73	Female	1
EE62	66	Female	3
EE64	81	Male	1
EE71	82	Female	2
EE72	68	Female	2
EE74	88	Female	1
EE75	74	Female	3
EE79	68	Female	3
EE83	74	Female	3
EE89	71	Female	1
EE90	90	Male	1
EE91	89	Female	1
EE95	66	Female	4
EE102	65	Female	3
EE107	65	Female	1
EE108	71	Male	1
EE113	66	Female	1

Groups: 1: Modulators of GABA receptors; 2: Modulators of serotonin availability; 3: Combination of groups 1 and 2; 4: Sleep regulators.

### 3.2. 16S taxonomy from fecal samples

A total 7,050,645 reads were sequenced from fecal samples, 18.23% of which were removed after quality check and host filtering, obtaining an average of 57,086 reads per sample (maximum length = 109,272, minimum length = 12,168, total number reads = 5,822,728). Taxonomic annotation showed two phyla with main representation in the EE cohort: Firmicutes (48.92%) and Bacteroidota (40.76%). Other phyla with lower representation included: Proteobacteria (4.25%), Actinobacteriota (2.82%), Verrucomicrobiota (1.31%), Desulfobacterota (0.70%), Cyanobacteria (0.15%), and Synergistota (0.12%).

**Figure 1:**
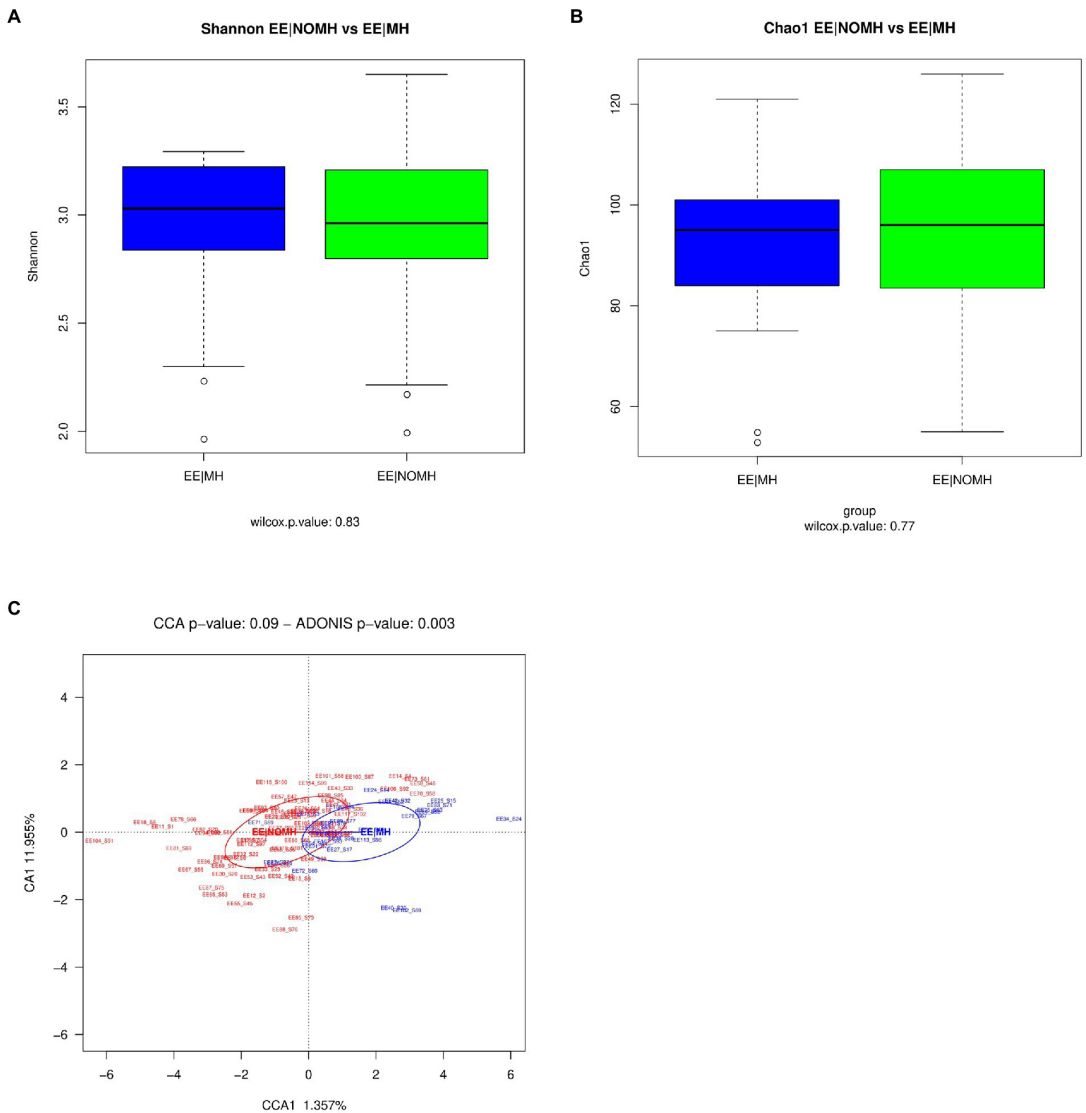
16S alpha and beta diversity of fecal samples. **(A)** Shannon diversity index and **(B)** richness estimator Chao1 analysis between EE|MH and EE|NOMH groups. **(C)** Canonical Correspondence Analysis (CCA) of EE|MH (blue) and EE|NOMH (red) groups at genus level.

**Figure 2:**
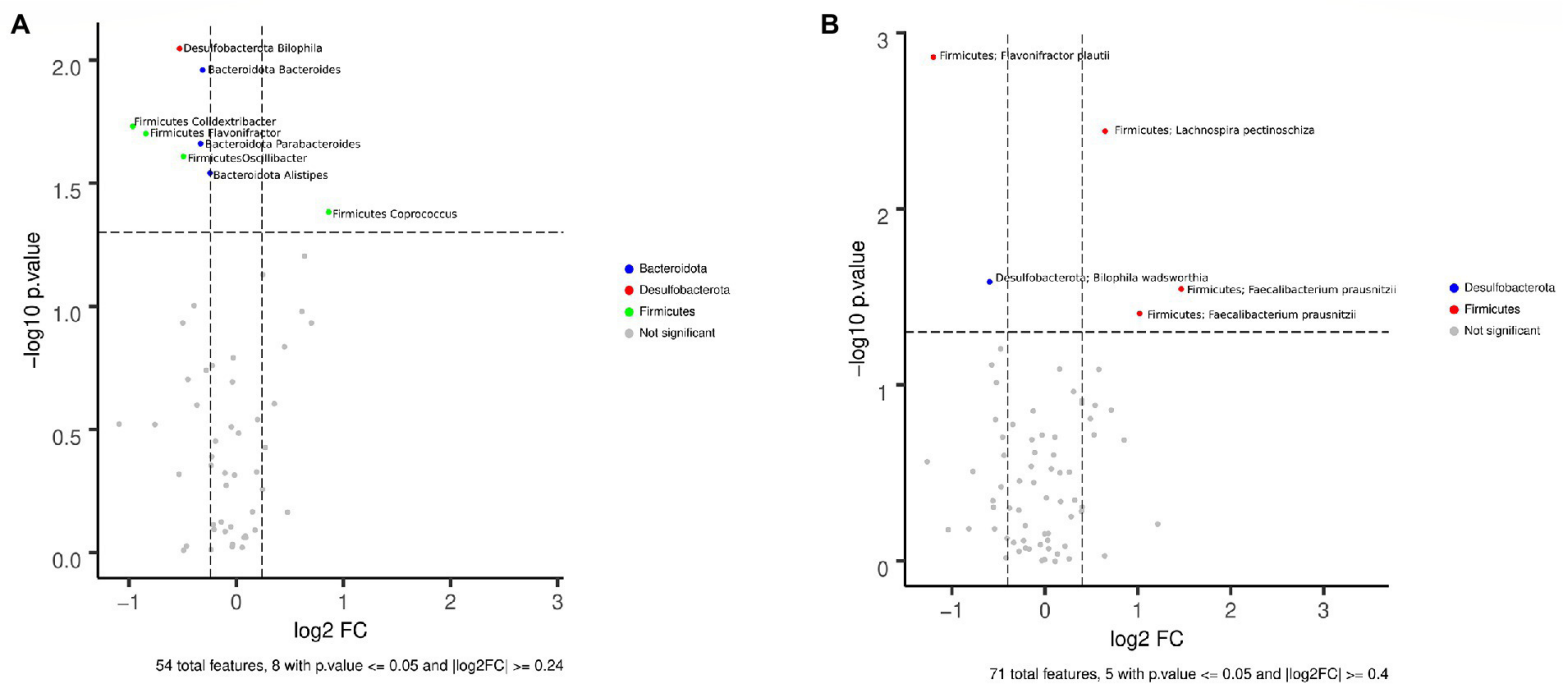
Volcano plots showing the differential abundance of **(A)** genera and **(B)** ASVs between EE|MH (right) and EE|NOMH (left) groups in fecal samples.

Alpha diversity analyses at genus and ASV levels showed that Shannon and Chao indexes were not statistically significant between EE|MH and EE|NOMH groups (Figures 1A,B). However, regarding beta diversity, the distribution of genera and ASV in the two groups was statistically significant (Adonis test, value of *p* = 0.003 and 0.038, respectively; Figure 1C). Because the EE/NOMH group has a clearly higher number of individuals than the EE/NOMH group (73 versus 28 volunteers), the analysis was repeated three times, each time choosing a group of 30 EE/NOMH individuals at random, in order to avoid bias due to the difference in the members of each group. In the three comparisons, the result was statistically significant with value of *p*s of 0.04, 0.021, and 0.002, respectively. The Wilcoxon non-parametric test also showed statistically significant differences (value of *p* < 0.05) between the two groups in the following eight genera (Figure 2A): *Bilophila, Bacteroides*, *Colidextribacter*, *Flavonifractor*, *Parabacteroides*, *Oscillibacter*, *Alistipes*, and *Coprococcus* and in five ASVs (Figure 2B), which correspond to the species *Flavonifractor plautii, Bilophila wadsworthia, Lachnospira pectinoschiza*, and two with *Faecalibacterium prausnitzii*. Of these, the genus *Coprococcus* and the ASVs corresponding to the species *Flavonifractor plautii* and *Bilophila wadsworthia* were more abundant in the EE|NOMH group.

### 3.3. 16S taxonomy from saliva samples

**Figure 3:**
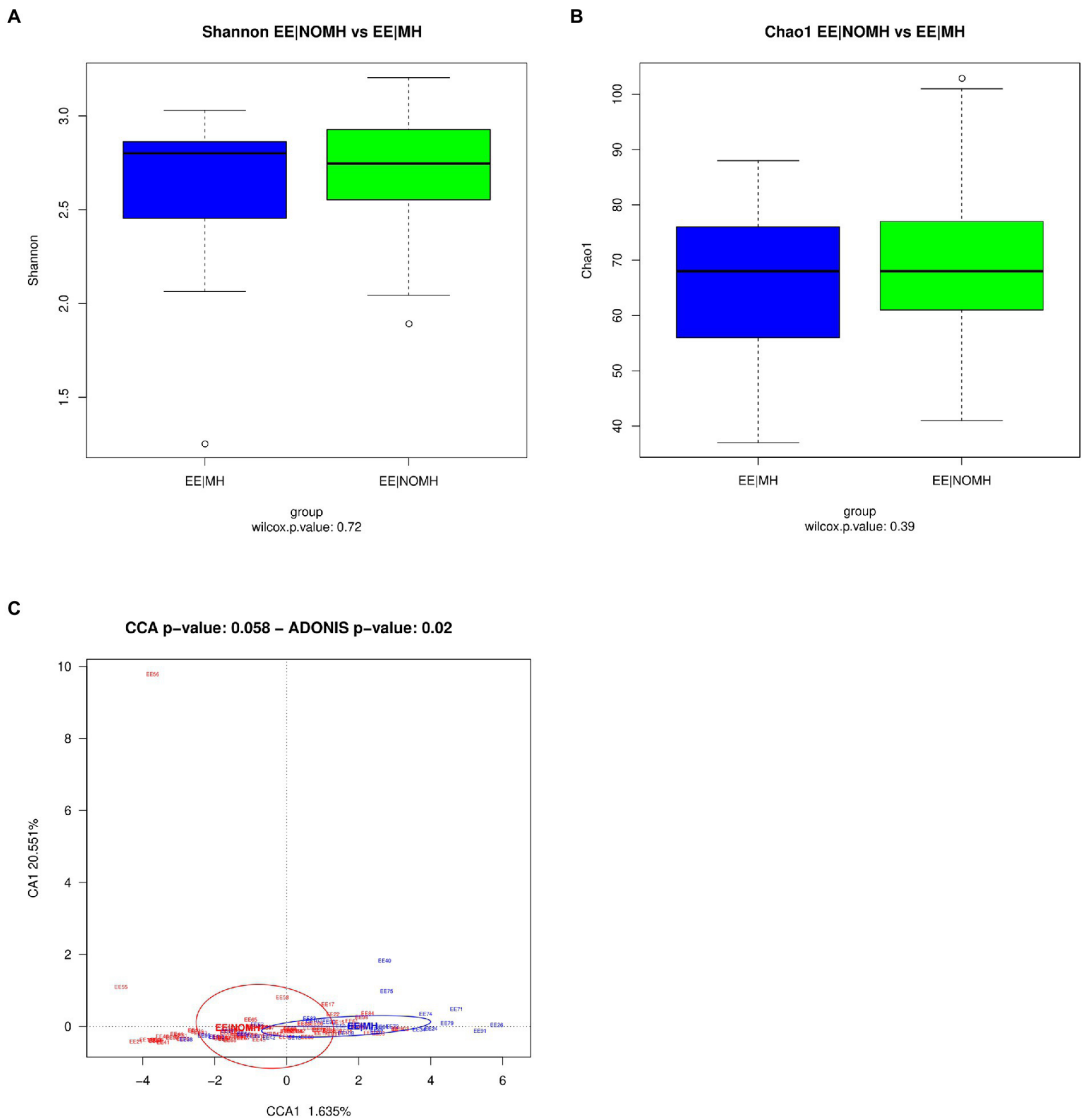
16S alpha and beta diversity of saliva samples. **(A)** Shannon diversity index and **(B)** richness estimator Chao1 analysis between EE|MH and EE|NOMH groups. **(C)** Canonical Correspondence Analysis (CCA) of EE|MH (blue) and EE|NOMH (red) groups at genus level.

**Figure 4:**
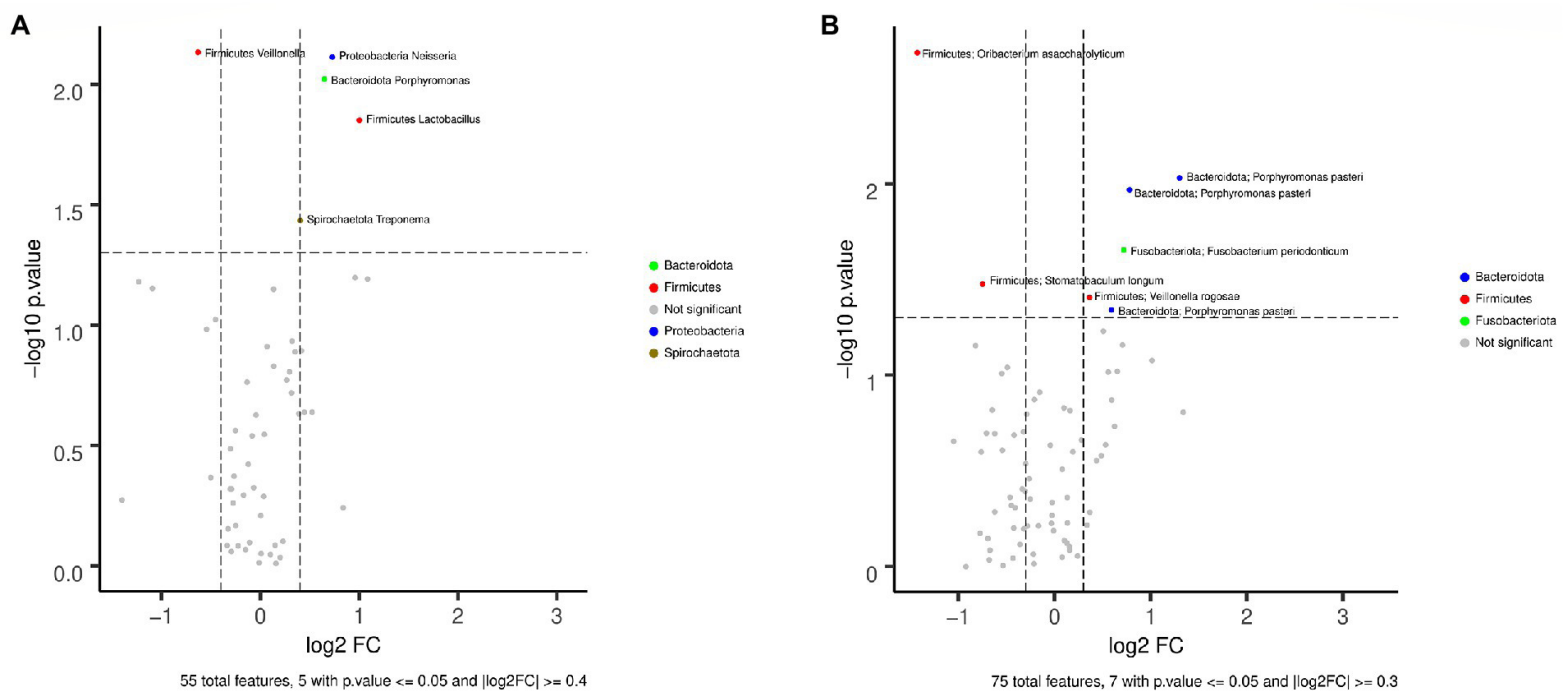
Volcano plots showing the differential abundance of **(A)** genera and **(B)** ASVs between EE|MH (right) and EE|NOMH (left) in saliva samples.

A total of 9,050,887 reads were sequenced from saliva samples, 24.47% of which were removed after quality check and host filtering, obtaining an average of 68,358.57 reads per sample (maximum length = 488,613, minimum length = 28,571, total number of reads = 6,835,857). Taxonomic annotation showed that the most represented phyla were Firmicutes (30.22%), Bacteroidota (28.91%), and Proteobacteria (20.48%), and other phyla with lower representation that included Fusobacteriota (8.15%), Actinobacteriota (5.82%), Patescibacteria (2.97%), Campilobacterota (2.21%), and Spirochaetota (0.83%). Similar results to those obtained with fecal samples were detected for the alpha diversity at the genus and ASV levels of saliva samples. Shannon and Chao indexes were not significantly different with *p* values >0.05 between EE|MH and EE|NOMH (Figures 3A,B). However, significant differences were found in the beta diversity between groups (Adonis test value of *p* = 0.02) (Figure 3C). We identified statistically significant differences (Wilcoxon test) for five genera (Figure 4A): *Veillonella*, Neisseria, *Porphyromonas*, *Lactobacillus*, and *Treponema*, and five ASV (Figure 4B), which corresponded to the species *Oribacterium asaccharolyticum, Stomatobaculum longum, Fusobacterium periodonticum, Veillonella rogosae*, and *Porphyromonas pasteri*. Only the genus *Veillonella* and the ASV corresponding to *Oribacterium asaccharolyticum* and S*tomatobaculum longum* were more abundant in EE|MH, while the rest were more abundant in EE|NOMH.

### 3.4. Correlation analysis between gut and saliva microbiota

**Figure 5:**
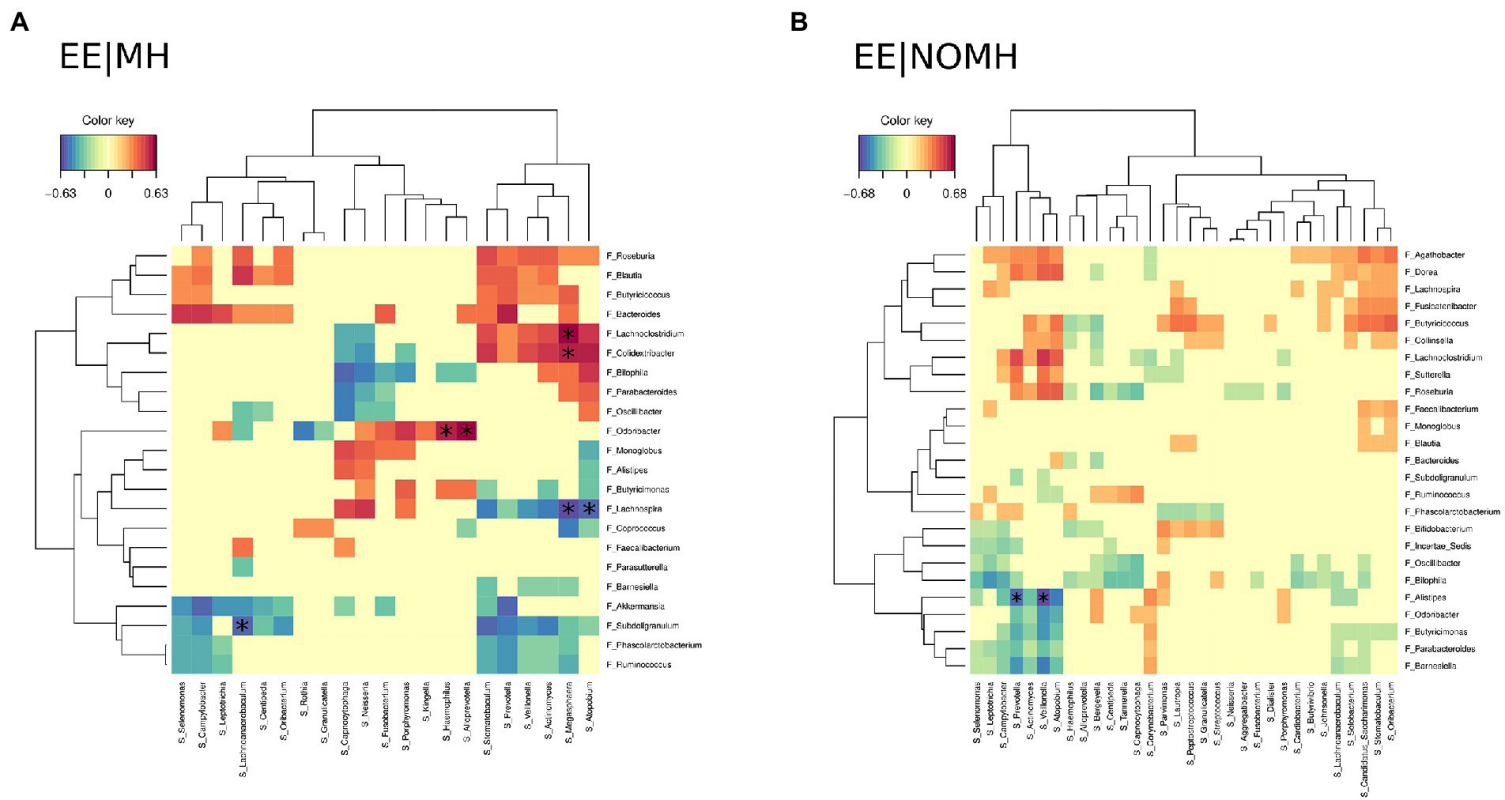
Heatmaps charts showing the correlations between gut and saliva microbiota in **(A)** EE|MH and **(B)** EE|NOMH groups.

Correlation analysis between the gut and the saliva microbiota at genus level was performed using the Mixomics single omic approach. The correlation analyses showed differences between both groups. In the EE|MH group the genus *Lachnospira* (gut) with the genera *Megasphaera* and *Atopobium* (saliva) and the genus *Subdoligranulum* (gut) with the genus *Lachnoanaerobaculum* (saliva) showed significant negative correlations, while the genus *Odoribacter* (gut) with the genera *Alloprevotella* and *Haemophilus* (saliva) and the genera *Lachnoclostridium*, and *Colidextribacter* (gut) with the genus *Megasphaera* (saliva) showed positive correlation (Figure 5A). By contrast, the genus *Alistipes* (gut) had significant negative correlation with the genera *Veillonella* and *Prevotella* (saliva) in the EE|NOMH group (Figure 5B).

### 3.5. Functional orthologs analysis from metagenome data of fecal samples

A total 554,768,576 reads were sequenced, 19.09% of which were removed after quality check and host filtering, obtaining an average of 4,444,094.52 reads per sample (maximum length = 15,434,500, minimum length = 924,728, number of total reads = 448,853,547); of these 270,608,848 were correctly assigned to KEGG Orthology (KO) categories (maximum number of reads assigned per sample = 10,033,384; minimum number of reads assigned per sample = 330,178).

**Figure 6:**
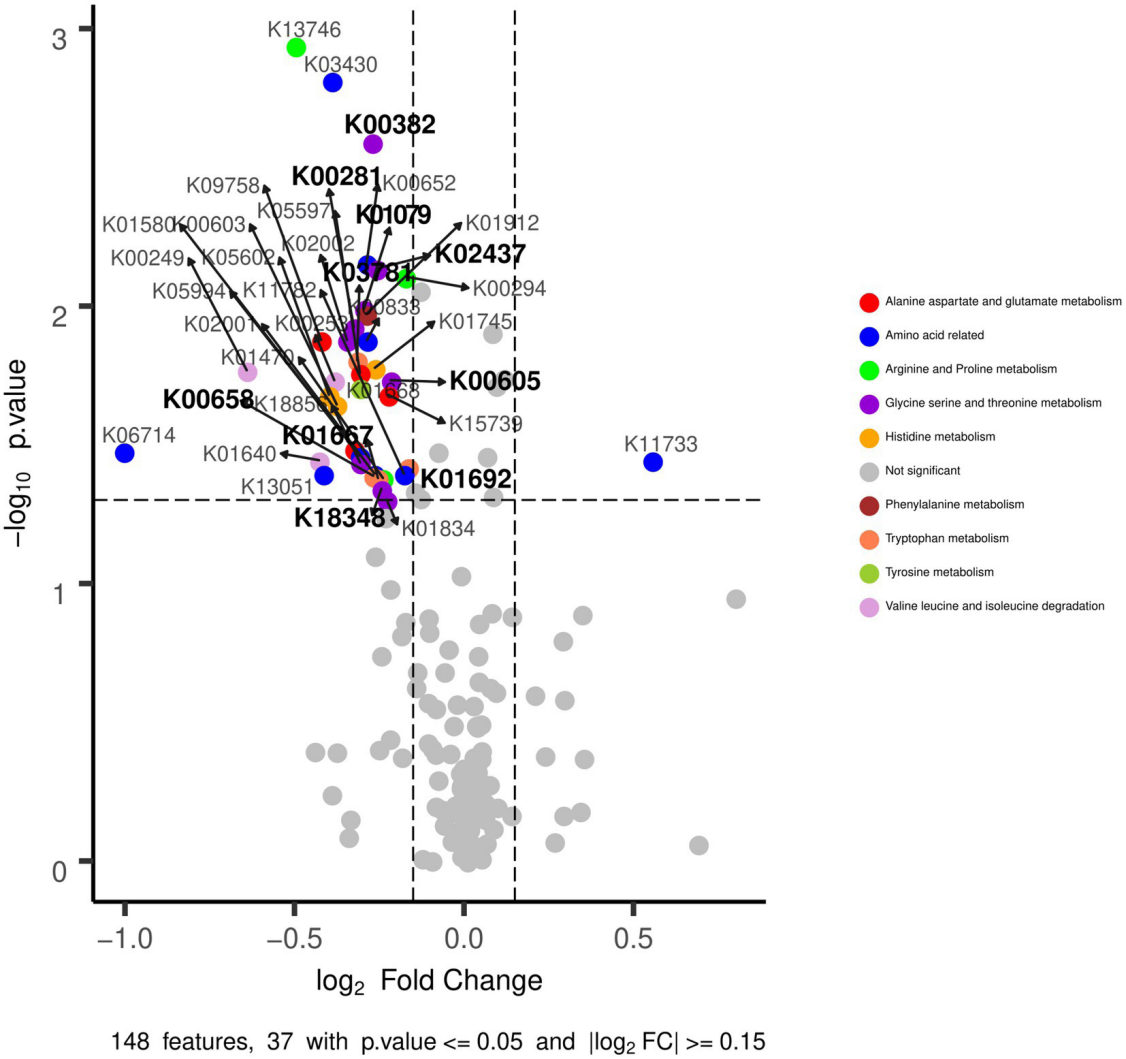
Significant differences in KEGG categories, in bold those related to tryptophan and serine metabolism between EE|NOMH (right) and EE|MH (left) groups.

No significant differences were found in the CCA analysis between EE|MH and EE|NOMH for KO categories (Adonis test value of *p* = 0.24; Supplementary Figure 1). However, the Wilcoxon test identified 382 KO categories that showed significant differences (value of *p* < 0.05). It is worth mentioning that five are involved in tryptophan metabolism (K00382, K03781, K01692, K00658, and K01667) (see Supplementary Figure 2) and six in serine metabolism (K00382, K02437, K01079, K00281, K00605, and K18348/K12235). Serine is used by bacteria to convert indole into tryptophan- (see Supplementary Figure 3), which were higher in EE|MH than in EE|NOMH (Figure 6). Furthermore, 19 KO categories involved in the synthesis of metabolic products related to GABA production was higher in the EE|MH group (K13746, K03474, K00294, K00175, K01425, K03473, K09758, K05275, K00174, K17865, K05597, K01580, K00262, K01640, K00634, K01692, K13051, K01470, and K09472). Three of these KO categories correspond to the arginine and proline metabolism pathway (K00294, K01470, and, K09472), five to the alanine aspartate and glutamate metabolism (K00262, K00294, K01425, K01580, and K05597), seven to the butanoate metabolism (K00174, K00175, K00634, K01580, K01640, K01692, and K17865), and three to the vitamin B6 metabolism (K03473, K03474, and K05275) which, as a co-factor, is also involved in the biosynthesis and catabolism of amino acids and neurotransmitters like GABA (Table 2). Finally, two more KO categories were shared by the arginine and proline metabolism and the alanine, aspartate, and glutamate metabolism pathways (K00294) and by the alanine, aspartate, and glutamate metabolism, and the butanoate metabolism pathways (K01580). The genus contribution to these KOs was obtained using taxonomy information from metagenomic data through Kaiju. The genera *Bacteroides* and *Alistipes* were the most representative in most of the KOs analyzed. It is noteworthy that the percentage of the *Bacteroides* contribution was higher in the EE|MH group in all but one KO and that the genus *Alistipes* had a higher contribution in most of the KOs in the EE|NOMH group (Table 2).

**Table 2:** Significantly different KO categories and the genera with the highest contribution to them in both groups.

KO	*p*-value	Pathway	Most abundant	Genera with the highest contribution in EE|MH	Genera with the highest contribution in EE|NOMH
K03781	0.016	Tryptophan metabolism	EE|MH	*Bacteroides* (45.5%) *Alistipes* (21.6%)	*Bacteroides* (36%) *Alistipes* (24%)
K00658	0.041	Tryptophan metabolism	EE|MH	*Bacteroides* (57.4%)	*Bacteroides* (52.3%)
K01667	0.042	Tryptophan metabolism	EE|MH	*Bacteroides* (29%) *Alistipes* (23.1%)	*Bacteroides* (22.6%) *Alistipes* (23.3%)
K02437	0.007	Glycine, serine and threonine metabolism	EE|MH	*Bacteroides* (31.5%) *Alistipes* (9.9%)	*Bacteroides* (27.1%) *Alistipes* (11.5%)
K01079	0.01	Glycine, serine and threonine metabolism	EE|MH	*Bacteroides* (36.8%) *Alistipes* (9.3%)	*Bacteroides* (34.5%) *Alistipes* (12.4%)
K00281	0.011	Glycine, serine and threonine metabolism	EE|MH	*Bacteroides* (43%) Alistipes (12.3%)	*Bacteroides* (39.7%) *Alistipes* (12.4%)
K00605	0.018	Glycine, serine and threonine metabolism	EE|MH	*Bacteroides* (46.7%) *Alistipes* (14.3%) *Prevotella* (6.2%)	*Bacteroides* (38.1%) *Alistipes* (13.1%) *Prevotella* (9.4%)
K18348 K12235	0.046	Glycine, serine and threonine metabolism	EE|MH	*Bacteroides* (16.1%) *ParaBacteroides* (6.2%) *Faecalibacterium* (6.2%)	*Bacteroides* (14.1%) *ParaBacteroides* (8.7%)
K13746	0.001	Arginine and proline metabolism	EE|MH	*Bacteroides* (60%)	*Bacteroides* (40%) *Roseburia* (20%)
K01470	0.042	Arginine and proline metabolism	EE|MH	*Bacteroides* (36.6%)	*Bacteroides* (34.8%)
K09472	0.049	Arginine and proline metabolism	EE|MH	*Bilophila* (16.7%) *Enterobacter* (16.7%) *Escherichia* (16.7%)	not assigned
K01425	0.009	Alanine, aspartate and glutamate metabolism	EE|MH	*Bacteroides* (41.6%) *Alistipes* (8.1%)	*Bacteroides* (31.6%) *Alistipes* (9.6%)
K09758	0.013	Alanine, aspartate and glutamate metabolism	EE|MH	*Bacteroides* (47.5%)	*Bacteroides* (43.7%)
K05597	0.017	Alanine, aspartate and glutamate metabolism	EE|MH	*Bacteroides* (62%) *Sutterella* (11%) *Phascolarctobacterium* (5.8%)	*Bacteroides* (59.8%) *Sutterella* (5%) *Phascolarctobacterium* (6.3%)
K00262	0.033	Alanine, aspartate and glutamate metabolism	EE|MH	*Bacteroides* (23.1%) *Faecalibacterium* (6.1%) *Alistipes* (4.3%)	*Bacteroides* (16.9%) *Faecalibacterium* (6.1%) *Alistipes* (4.8%)
K13051	0.04	Alanine, aspartate and glutamate metabolism	EE|MH	*Bacteroides* (68.4%) *Bifidobacterium* (5.3%)	*Bacteroides* (64.4%) *Bifidobacterium* (4.4%)
K00175	0.009	Butanoate metabolism	EE|MH	*Bacteroides* (34.6%) *Alistipes* (8.4%) *Prevotella* (7.5%)	*Bacteroides* (28.7%) *Alistipes* (8.4%) *Prevotella* (8.9%)
K00174	0.015	Butanoate metabolism	EE|MH	*Bacteroides* (38%) *Alistipes* (9.8%) *Prevotella* (6.3%)	*Bacteroides* (33.6%) *Alistipes* (11%) *Prevotella* (8%)
K17865	0.016	Butanoate metabolism	EE|MH	*Bacteroides* (33.3%) *Alistipes* (55.6%)	*Bacteroides* (5.9%) *Alistipes* (52.9%)
K01640	0.036	Butanoate metabolism	EE|MH	*Faecalibacterium* (46.7%) *Phascolarctobacterium* (26.7%)	*Faecalibacterium* (9.1%) *Phascolarctobacterium* (45.5%) *Cloacibacillus* (9.1%)
K00634	0.037	Butanoate metabolism	EE|MH	*Bacteroides* (32.3%) *Alistipes* (11.1%) *Prevotella* (5.5%)	*Bacteroides* (26.5%) *Alistipes* (9.4%) *Prevotella* (8.7%)
K00170	0.042	Butanoate metabolism	EE|NOMH	*Clostridium* (29.2%) *Roseburia* (12.5%) *Sutterella* (12.5%)	*Clostridium* (62.7%) *Sutterella* (7.2%)
K03474	0.003	Butanoate metabolism	EE|MH	*Bacteroides* (46.2%) *Alistipes* (8.8%)	*Bacteroides* (38.3%) *Alistipes* (10.4%) *Prevotella* (10.4%)
K03473	0.013	Butanoate metabolism	EE|MH	*Bacteroides* (31.8%) *Alistipes* (11.5%) *Prevotella* (6.5%)	*Bacteroides* (55.6%) *Alistipes* (10.6%) *Prevotella* (8.4%)
K05275	0.014	Butanoate metabolism	EE|MH	*Bacteroides* (36%)	*Bacteroides* (31.6%) *Prevotella* (5.8%)
K08681	0.039	Butanoate metabolism	EE|NOMH	*Roseburia* (15.9%) *Butyrivibrio* (7.9%) *Eubacterium* (7.9%)	*Prevotella* (25.6%) *Butyrivibrio* (6.6%)
K00382	0.0026	Tryptophan metabolism Glycine, serine and threonine metabolism	EE|MH	*Bacteroides* (56.6%) *Alistipes* (6.5%)	*Bacteroides* (49.1%) *Alistipes* (8%)
K01692	0.038	Tryptophan metabolism Butanoate metabolism	EE|MH	*Clostridium* (14.3%) *Oscillibacter* (14.3%) *Sutterella* (14.3%)	*Clostridium* (7.1%) *Coprococcus* (7.1%) *Oscillibacter* (7.1%) *Faecalibacterium* (7.1%) *Phascolarctobacterium* (7.1%) *Sutterella* (7.1%) *Klebsiella* (7.1%)
K00294	0.007	Arginine and proline metabolism Alanine, aspartate and glutamate metabolism	EE|MH	*ParaBacteroides* (20%) *Coprococcus* (6.7%) *Butyricimonas* (6.7%)	*ParaBacteroides* (20%) *Coprococcus* (8%) *Odoribacter* (8%)
K01580	0.032	Alanine, aspartate and glutamate metabolism Butanoate metabolism	EE|MH	*Bacteroides* (41.5%) *Alistipes* (16.7%)	*Bacteroides* (34.7%) *Alistipes* (17.5%)

### 3.6. Analysis of KEGG metabolic pathways

**Figure 7:**
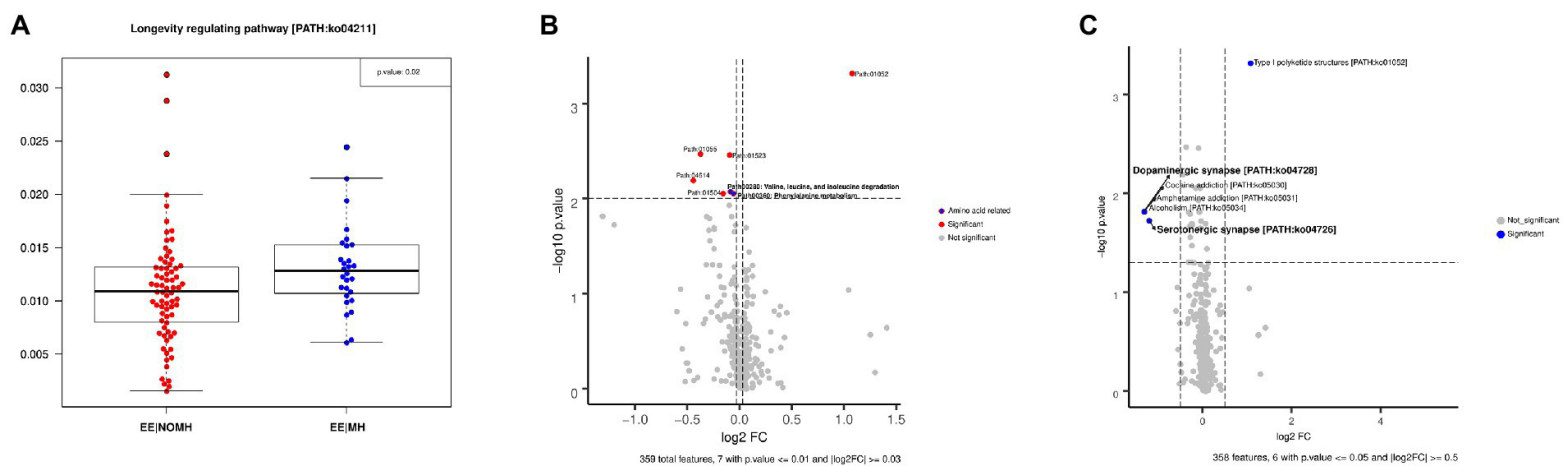
**(A)** Differences in the longevity regulating pathway between EE|NOMH (red) and EE|MH (blue). **(B)** Significant differences in the metabolism of amino acids between EE|NOMH (right) and EE|MH (left). **(C)** Significant differences in neuronal synapses between EE|NOMH (right) and EE|MH (left) groups.

CCA analysis carried out between EE|MH and EE|NOMH showed no statistically significant differences in KEGG pathways (Adonis test *p* − value = 0.25; see Supplementary Figure 4). However, 29 KEGG pathways showed significant differences in the Wilcoxon test. Interestingly, considering that both groups consisted of individuals over the age of 65, the pathway regulating longevity was significantly higher in EE|MH than in EE|NOMH (path 04211, value of *p* = 0.02; Figure 7A). In addition, two amino acid metabolism pathways also showed higher abundance in the EE|MH group: valine, leucine, and isoleucine degradation (path 00280, value of *p* = 0.0084) and phenylalanine metabolism (path: 00360, value of *p* = 0.0088; Figure 7B). Finally, the other two significant KEGG pathways related to the CNS and tryptophan metabolism were higher in the EE|MH group, the dopaminergic synapse (path: 04728 value of *p* = 0.015) and serotoninergic synapse (path 04726, value of *p* = 0.019; Figure 7C).

### 3.7. Robustness analysis of samples

EE|MH and EE|NOMH groups showed no differences in either attenuation (Mann–Whitney test value of *p* = 0.072) or buffering (Mann–Whitney test *p* value = 0.15) in fecal samples. Attenuation and buffering for each individual are shown in Supplementary Table 2. In addition, values of attenuation and buffering for each individual within each group (Supplementary Figure 5A) are not correlated after applying Pearson’s correlation coefficient. In some cases, individual pathways start from a common precursor, or produce a common product, but they can also have other relationships. Superpathways can have individual reactions due to their components in addition to other pathways. Moreover, distribution curves of attenuation and buffering (Supplementary Figures 5B,C, respectively) were similar for both groups, controls and treated individuals. Similar results to those observed in fecal samples were observed for saliva. Attenuation and buffering for each individual saliva sample are shown in Supplementary Table 3. Attenuation (Mann–Whitney test, *p* value = 0.41) and buffering (Mann–Whitney test, *p* value = 0.95 for Buffering) were not significant between groups. Moreover, values of attenuation and buffering for each individual within each group (Supplementary Figure 6A) did not correlate after applying Pearson’s correlation coefficient. Furthermore, distribution curves of attenuation and buffering (Supplementary Figures 6B,C, respectively) were also similar for both groups, controls and treated.

Of the 20 main superpathways, most will have an additional parent class within the pathway ontology to define their biological role. Statistical differences for attenuation were found for fecal and saliva samples in superpathways for both groups (Supplementary Figures 7A,B, respectively). In fecal samples only in superpathway cell motility (lower attenuation in treated group, value of *p* in Kruskal–Wallis test 0.0428) while in saliva samples, we found differences in attenuation for four superpathways (higher for treated group in superpathways for lipid metabolism and translation and lower in metabolism of terpenoids and polyketides, and cell growth and death). In case of buffering, no differences were found in fecal samples (Supplementary Figure 7C), while differences were recorded in only four superpathways in saliva samples: lipid metabolism, metabolism of other amino acids and folding, sorting and degradation (lower for treated group) and, finally, metabolism of terpenoids and polyketides (higher in treated group; Supplementary Figure 7D).

## 4. Discussion

Tryptophan is an essential amino acid for protein synthesis, and the least abundant amino acid in proteins and cells (Gao et al., 2020). Certain bacterial products of tryptophan metabolism, including serotonin, indolic compounds, and kynurenines, can interact with the gut-brain axis and the CNS of the host, thereby modulating physiology (Agus et al., 2018). Changes affecting the gut-brain axis are thought to be connected to a number of neurological disorders, such as Parkinson’s disease, Autism spectrum disorder, and Alzheimer’s disease, as well as some gastrointestinal (GI) disorders, such as irritable bowel syndrome and obesity, and even some psychological disorders, such as depression, anxiety, and insomnia (Liang et al., 2018; Richards et al., 2021). Other authors have focused on the role of the microbiota in the development of mental health-related conditions, discussing that conditions characterized by acute or chronic inflammation, depression, decreased quality of life or cognitive impairment are related to the metabolic alteration of amino acid precursors of neurotransmitters, such as tryptophan and phenylalanine among others Strasser et al., 2017.

Around 90–95% of available tryptophan goes through the KP, 1–2% of it forms 5-HT and melatonin through the serotonin pathway, and 4–6% is metabolized into indole and other indolic derivates by bacteria (Gao et al., 2018), that can be transferred across the blood–brain barrier to reduce neuroinflammation Cox and Weiner, 2018. The microbiota plays an important role, for instance, it is crucial for the gut’s amino acid metabolism, which has an impact on neuroinflammatory illnesses. The ability of the microbiota to access gut-brain signaling pathways and modify the host’s behavior depends on bidirectional communication along the gut-brain axis. TPH2 is the protein that catalyzes the first step in serotonin biosynthesis from tryptophan in the brain. An imbalance in serotonin levels has been widely associated with neuropsychiatric disorders such as depression and anxiety (Pelosi et al., 2015). Shishkina et al. showed that TPH2 expression increases in the midbrain in animal models of depression treated with antidepressants (Shishkina et al., 2007). In our study, the volunteers with depression were also taking antidepressant medication, which might increase the abundance of genera strongly correlated with TPH2, such as *Bilophila* (Liu G. et al., 2020). In fact, the genus *Bilophila* proved significantly higher in the EE|MH group. These bacteria are reported to be significantly increased in anhedonia (loss of pleasure) in mouse models. Anhedonia is one of the two core symptoms of depression (Yang et al., 2019) and was also found to be increased in a mouse model of depression, subjected to chronic unpredictable mild stress (Zhang M. et al., 2021). *Bilophila* has also been described as positively correlated with tryptophan hydroxylase 2 (TPH2) gene expression (Liu G. et al., 2020).

Over 90% of the whole tryptophan is metabolized through the KP. IDO and tryptophan 2, 3-dioxygenase (TDO) are the enzymes that catalyze the first step of tryptophan metabolism in this pathway (Maes et al., 2011). On the one hand, TDO activation is normally stable and is regulated by tryptophan availability (Gao et al., 2020). On the other hand, IDO is induced by interferon-gamma (IFN-*γ*) and tumor necrosis factor-alpha (TNF-*α*) among other pro-inflammatory cytokines, and its activation is correlated with the intensity of depressive symptoms (Höglund et al., 2019; Gao et al., 2020; Chen et al., 2021). IDO activation by inflammation caused by bacteria such as *Flavonifractor* and *Alistipes* and promoting KYN formation through KP can decrease tryptophan availability, negatively impacting serotonin synthesis and neurotransmission. *Flavonifractor* and *Alistipes* were significantly higher in the mental-health treatment group (EE|MH). *Flavonifractor* has previously been reported as higher in individuals with major depressive disorder (Jiang et al., 2015; Valles-Colomer et al., 2019), generalized anxiety disorder (Jiang et al., 2015), affective disorders (Coello et al., 2019), and bipolar disorder (Lindseth et al., 2015; Wang et al., 2021). *Flavonifractor* has also been described as a pro-inflammatory genus and studies show a negative association between this genus and quality of life scores (Jiang et al., 2018). Parker et al. (2020) and Jiang et al. (2015) also described *Alistipes* to be higher in patients with depression (Jiang et al., 2018; Parker et al., 2020). This genus is believed to be associated with stress, fatigue syndrome, and depressive disorders through inflammatory pathways (Naseribafrouei et al., 2014).

*Bacteroides* were also significantly higher in the EE/MH mental-health treatment group. The role of *Bacteroides* in mental health is highly controversial, with some authors observing the genus to be lower in patients suffering from mental health disorders (Jiang et al., 2015), while others find it to be higher in this group (Yang et al., 2019). This genus has previously been studied for its ability to produce cytokines and its role in inflammation, as gut inflammation has a clear association with depression (Schiepers et al., 2005; Dantzer, 2009). By contrast, *Flavonifractor* is reported to be higher in patients with remitted geriatric depression (Lee et al., 2022), which might explain why it is higher in the EE|MH group, where elderly subjects are medicated for mental health. In this case, the medication might be responsible for remission.

During aging, elderly individuals suffer from systematic inflammation and, as stated above, mental illness is generally associated with an inflammatory state of the patient. Oral health is also influenced by aging and inflammation, with an increased prevalence of periodontal disease (Clark et al., 2021). Several studies suggest that some psychiatric diseases, such as Alzheimer’s or bipolar disorder, are related to leakage of pro-inflammatory oral bacteria triggering neuroinflammation (Leira et al., 2017). Furthermore, mental health issues such as anxiety and depression are related to a decrease in oral hygiene and dental check-ups (Anttila et al., 2006; Okoro et al., 2012; Simpson et al., 2020). Periodontal diseases (mainly periodontitis and gingivitis) are caused by bacterial-induced inflammation. *Porphyromonas* is a well-known periodontal pathogen whose virulence factors cause deregulation in inflammatory and immune responses of the host (Mysak et al., 2014; Leira et al., 2017). Studies of Alzheimer’s disease show inflammatory cytokines such as TNF-*α*, IL-1, IL-6, and IL-8 are released from the host cells that have been infected with *Porphyromonas* (Mei et al., 2020). Similarly, *Treponema denticola* is known to cause gingivitis in cases of oral dysbiosis, despite being a normal component of human oral microbiota (Simpson et al., 2020).

*Porphyromonas* and *Treponema* were both higher in our EE|NOMH group saliva. Both bacteria can form synergistic biofilms and are positively associated with chronic periodontitis and severe periodontal disease (Ng et al., 2019). The genus *Veillonella*, which was found to be higher in the EE|MH group, was previously correlated with anti-inflammatory mediators and maintains oral pH by metabolizing lactate into weaker acids (Rosier et al., 2018). In the case of oral microbiota, we also observed the influence of mental health medicine in restoring elderly participants to a healthier state, as the medicated EE|MH group had significantly lower abundances of these pro-inflammatory genera.

Correlation analysis of both oral and intestinal microbiota, showed similar results. In the EE|NOMH group, the genus *Alistipes* from the gut was negatively correlated with the oral genera Veillonella and *Prevotella*. As stated above, *Alistipes* is a pro-inflammatory genus that has previously been correlated with mental health problems while oral *Veillonella* and *Prevotella* were negatively correlated with pro-inflammatory markers, *Prevotella* has been even negatively associated with distress (Kohn et al., 2020). Meanwhile, in the EE|MH group the gut genus *Lachnospira* showed a negative correlation with the oral genera *Megasphaera* and *Atopobium*. Previous studies report *Lachnospira* to be lower in animal models of depression and stress (Flux and Lowry, 2020), and in a cohort of patients suffering major depressive disorder (Rosier et al., 2018). By contrast, *Megasphaera* and *Atopobium* are found to be higher in cohorts with mental health disorders (McGuinness et al., 2022). Similarly, the oral genus *Lachnoanaerobaculum* and the intestinal genus *Subdoligranulum* showed significant negative correlations in the EE|MH group. Liu R. T. et al. (2020) reported that the abundance of *Subdoligranulum* was reduced in subjects who had more severe symptoms of depression (Liu R. T. et al., 2020), while Wang et al. described an augmented abundance of *Lachnoanaerobaculum* in depression and anxiety (Wang et al., 2022).

On the other hand, *Odoribacter* in gut microbiota with the oral genera *Alloprevotella* and *Haemophilus* had a positive correlation in the EE|MH group, all three of these genera are related with bad health. *Odoribacter* is one of the gut microbes associated with mental health issues, including major depressive disorder (Zhang M. et al., 2021). Oral *Alloprevotella* is described to be involved in periodontal disease (Sun et al., 2020) and *Haemophilus* is a well-known oral pathogen (Nørskov-Lauritsen, 2014). A similar positive correlation was obtained in the EE|MH group between *Lachnoclostridium*, and *Colidextribacter* from the intestinal microbiota with the oral genus *Megasphaera* that, as stated above, is elevated in mental health disorders. *Lachnoclostridium* has been associated with higher depressive symptoms in an induced animal model of depression (Zhang Y. et al., 2021), while *Colidextribacter* was associated with a positive response to antidepressant treatment in a mouse model of depression (Duan et al., 2021). Together these results again indicate that the mental-health treatment the EE|MH group may be restoring the microbiota to a healthier state, even though some genera related with mental health disorders are still present.

*Parabacteroides*, another genus involved in tryptophan metabolism, was significantly higher in the EE|MH group. Deng et al. (2021) showed that the genus plays an important role in tryptophan metabolism, where it has a strong correlation between the KP and depressive-like behavioral changes in a rat model of chronic restraint stress (Wu et al., 2014; Deng et al., 2021). Moreover, Li et al. (2016) described that a decrease in the abundance of *Parabacteroides* correlated with an improvement in the mood of adults.

Functional analysis of metagenome data showed five KEGG Orthology categories that are significantly higher in the EE|MH group and are related to tryptophan metabolism. Interestingly, tryptophanase (K01667) was markedly higher in the EE|MH group. This enzyme carries out the first step in the indolic pathway, transforming tryptophan into indole (Agus et al., 2018). Indole is a signaling molecule that can control bacteria antibiotic resistance, sporulation, and biofilm formation. It can also inhibit quorum sensing and modulate virulence factors (Agus et al., 2018). Indolic compounds are AhR ligands; AhR activation influences immune homeostasis *via* receptor anti-inflammatory effect by regulating intraepithelial lymphocytes and innate lymphoid cells (Li et al., 2011; Qiu et al., 2012; Jin et al., 2014). They are known to extend the health-span of several models of aging, such as *C. elegans*, *D. melanogaster*, and mouse (Sonowal et al., 2017).

Ruiz-Ruiz et al. (2020) showed the loss of the tryptophanase enzyme during aging and describe how the microbiota diminishes its ability to produce indole and tryptophan in old age, compromising the health status of the elderly (Ruiz-Ruiz et al., 2020). Our EE|NOMH group, comprising over 65-year-olds who are not taking mental-health medication, had a significantly lower abundance of tryptophanase. This would indicate that medication, such as antidepressants and anti-anxiolytic drugs, restore these individuals to a healthier state, which might also explain the significant difference in the longevity regulating pathway between the EE|MH and EE|NOMH groups.

GABA is the principal inhibitory neurotransmitter in the brain. It affects the control of homeostasis during stress and has been associated with mental health disorders such as anxiety and depression (Geuze et al., 2008). GABA and several other GABA analogs have been shown to have anxiolytic and hypnotic effects. Positive modulators to GABA receptors have been used to treat anxiety disorders and insomnia (Kalueff and Nutt, 2007). Classic mental-health treatments include benzodiazepines, these are positive allosteric modulators of GABA receptors (Sigel and Ernst, 2018).

*Oscillibacter*, which we found to be enriched in the EE|MH group, has valeric acid as its main metabolic product; this metabolite mimics GABA. Valeric acid can bind with the GABAa receptor, which explains the association between valeric acid-producing bacteria and depression (Naseribafrouei et al., 2014). Rong et al. (2019) reported similar results, finding an increase of this genus in treated patients suffering from major depressive disorder or bipolar disorder (Rong et al., 2019). Similarly, the GABA producing genus *Bacteroides* (Otaru et al., 2021) was also higher in the EE|MH group. The contribution of genera to the analyzed KOs showed that *Bacteroides* and *Alistipes* were the ones contributing most to the production of these KOs in the EE|MH group. It is noteworthy that these genera were also significantly higher in the EE|MH group.

The dopaminergic synapse, which includes alcoholism, the amphetamine addiction, and the cocaine addiction pathways, was higher in our EE|MH group. Dopamine is a neurotransmitter responsible for several functions in the body, including learning, memory, reward, and motor control. It has been implicated in psychiatric and psychological disorders (Ko and Strafella, 2012). Dopamine availability is higher in cocaine and amphetamine users, and the reward system in the brain was active in animal models of addiction (Di Chiara et al., 2004). The use of benzodiazepines as mental-health medication in the EE|MH group also explains the difference in synaptic pathways between both groups. Benzodiazepines are positive allosteric modulators of GABA receptors, and it has been suggested that the activation of GABA receptors enhances dopamine release (Kramer et al., 2020).

The results show that treatment-related changes in taxonomic composition of microbiota modifies robustness parameters, in other words, eventual changes in taxonomic composition modify functional capacity of the bacterial community, at least in some superpathway functions. However, it is important remark that this functional capacity is based only on the content of all genes of prokaryotic organisms living in microbiota, without considering the expression levels of every gene.

In the fecal microbiota, there are differences in taxonomic abundances in treated and not treated subjects, with a relevant impact on functional capacity and robustness for at least for some superpathways. For attenuation, differences were observed only in superpathway cell motility, while in buffering, no differences were found. Particularly interesting are the results for saliva samples, which show some differences in buffering. These were lower for at least three superpathways related with metabolism, which can induce variations in the metabolite landscape if alterations occur. These corresponded to the superpathway of lipid metabolism, superpathway of metabolism of terpenoids and polyketides and superpathway of metabolism of other amino acids. Also changes in attenuation were found (higher for superpathway of lipid metabolism and lower for superpathway of metabolism of terpenoids and polyketides). For the remaining superpathways not directly related with metabolism the differences observed could induce variations in cell division and growth of prokaryotic cells, without obvious consequences in community taxonomic composition dynamics, which could finally modify the values of those parameters of robustness.

Therefore, at least in oral microbiota, in-depth studies should address the relationship between changes induced in functional capacity by alteration or disturbances of the microbial community, and the medication to treat pathologies. In this respect, there are numerous references on the role of medication in Xerostomia related with salivary gland dysfunction, and oral diseases associated to diazepine (De Almeida et al., 2008) and other mental-health drugs (Koller et al., 2000; Arany et al., 2021) or medication commonly used by the elderly population (Leal et al., 2010).

Spain has an aging population, with 19.93% of the whole population over 65 years of age, in 2021. This age range corresponds to a higher risk of suffering mental health issues, reaching around 25% in 65-year-olds and up to 55% in 85-year-olds (Conselleria de Sanitat Universal i Salut Pública, 2020). It is important to identify the reasons underlying this increase in mental health issues in a population that registered 1,281 suicides in people over 65 in 2020 in Spain (Instituto Nacional de Estadistica, 2020). Here, we have demonstrated that there are significant differences in the microbiome composition and function of older people in the Comunidad Valenciana being medicated for mental health issues. Our results also indicated that the medication might help to recover the microbiome to a healthier state and aid patient remission by remodeling the gut microbiota and bacterial tryptophan metabolism.

## Data Availability

The datasets presented in this study are deposited in the European Nucleotide Archive repository (https://www.ebi.ac.uk/ena), accession number PRJEB56919.

## Footnotes

^1^ https://www.ine.es/

^2^ https://github.com/borenstein-lab/robustness
