# Correction: Influence of mental health medication on microbiota in the elderly population in the Valencian region

**DOI:** 10.3389/fmicb.2026.1861757

**Published:** 2026-05-13

**Authors:** Nicole Pesantes, Ana Barberá, Benjamí Pérez-Rocher, Alejandro Artacho, Sergio Luís Vargas, Andrés Moya, Susana Ruiz-Ruiz

**Affiliations:** 1 Fundación para el Fomento de la Investigación Sanitaria y Biomédica de la Comunitat Valenciana (FISABIO), València, Spain; 2 Consorcio de Investigación Biomédica en Red de Epidemiología y Salud Pública (CIBEResp), Madrid, Spain; 3 Instituto de Biología Integrativa de Sistemas (I2Sysbio), CSIC-Universitat de València, València, Spain; 4 Programa de Microbiología y Micología, Instituto de Ciencias Biomédicas, Facultad de Medicina, Universidad de Chile, Santiago, Chile

**Keywords:** aging, microbiota, gut-brain axis, mental health disorders, 16S rRNA gene sequencing, metagenomics, tryptophan

In the published article, there was a mistake in the **Funding** statement. The **Funding** statement for the Spanish Ministry of Science and Innovation was displayed as (PID2019-105969GB-I00). The correct statement is “Spanish Ministry of Science and Innovation PID2019-105969GB-I00 funded by MICIU/AEI /10.13039/501100011033”.

The correct **Funding** section is:

“The author(s) declared that financial support was received for this work and/or its publication. This research was funded by the Spanish Ministry of Science and Innovation PID2019-105969GB-I00 funded by MICIU/AEI/10.13039/501100011033 and supported by a grant (Programa Santiago Grisolía, GRISOLIAP/2019/080) from the Generalitat Valenciana”.

The original version of this article has been updated.

