# Supplementary material for: Influence of mental health medication on microbiota in the elderly population in the Valencian region
Source: Front Microbiol. 2023 Mar 16;14:1094071. doi: 10.3389/fmicb.2023.1094071 (PMC10062206; doi:10.3389/fmicb.2023.1094071)
Supplement: Supplementary file 1 [file Data_Sheet_1.PDF]

Supplementary Table 1. Information of the BMI, employment and tobacco and alcohol consumption of the volunteers over 65 years from the Valencian Community

ID	Group	BMI	Employment	Smoker	Habitual consumption of alcohol
**EE11**	EE|NOMH	22.7	Yes	No	No
**EE12**	EE|NOMH	20.7	No	No	Yes
**EE13**	EE|MH	18.0	No	Yes	No
**EE14**	EE|NOMH	22.1	No	No	Yes
**EE15**	EE|NOMH	22.8	No	Yes	No
**EE16**	EE|NOMH	30.2	No	No	No
**EE17**	EE|NOMH	31.5	No	No	Yes
**EE18**	EE|NOMH	29.8	No	No	No
**EE19**	EE|NOMH	28.5	No	Yes	No
**EE20**	EE|NOMH	25.4	No	No	No
**EE21**	EE|NOMH	23.8	No	No	Yes
**EE22**	EE|NOMH	27.1	No	No	No
**EE23**	EE|NOMH	24.2	No	No	No
**EE24**	EE|MH	31.4	No	No	Yes
**EE25**	EE|MH	25.8	No	No	No
**EE26**	EE|MH	27.3	No	No	No
**EE27**	EE|MH	24.7	No	No	No
**EE28**	EE|NOMH	20.5	No	No	No
**EE30**	EE|NOMH	24.7	No	No	No
**EE31**	EE|NOMH	24.9	No	No	No
**EE32**	EE|NOMH	29.9	No	No	No
**EE33**	EE|NOMH	25.3	Yes	Yes	No
**EE34**	EE|MH	23.4	No	Yes	No
**EE35**	EE|NOMH	22.6	No	No	No
**EE36**	EE|NOMH	32.0	No	No	No
**EE37**	EE|MH	24.5	No	No	No
**EE38**	EE|MH	22.1	No	No	No
**EE39**	EE|NOMH	33.8	No	Yes	Yes
**EE40**	EE|MH	25.0	No	No	No
**EE41**	EE|NOMH	34.4	N/A	N/A	N/A
**EE42**	EE|MH	22.9	No	No	No
**EE43**	EE|NOMH	29.4	No	No	No
**EE44**	EE|NOMH	31.5	No	No	Yes
**EE45**	EE|NOMH	30.6	No	No	No
**EE46**	EE|NOMH	27.3	No	No	No
**EE47**	EE|NOMH	26.8	No	No	No
**EE48**	EE|NOMH	19.4	No	No	No
**EE49**	EE|NOMH	24.7	No	No	No
**EE50**	EE|MH	26.5	N/A	N/A	N/A
**EE51**	EE|NOMH	22.0	No	No	No
**EE52**	EE|NOMH	21.0	No	No	Yes
**EE53**	EE|NOMH	26.6	Yes	No	Yes

**EE54**	EE|MH	24.8	Yes	No	Yes
**EE55**	EE|NOMH	24.5	N/A	N/A	N/A
**EE56**	EE|NOMH	30.9	No	No	No
**EE57**	EE|NOMH	29.4	No	No	No
**EE58**	EE|NOMH	30.0	No	No	No
**EE59**	EE|NOMH	31.1	No	No	No
**EE61**	EE|NOMH	32.0	No	Yes	No
**EE62**	EE|MH	29.4	No	Yes	No
**EE64**	EE|MH	24.4	No	No	No
**EE65**	EE|NOMH	30.9	No	No	No
**EE66**	EE|NOMH	23.7	No	No	No
**EE67**	EE|NOMH	27.0	No	No	No
**EE68**	EE|NOMH	24.5	No	No	No
**EE69**	EE|NOMH	24.0	No	No	No
**EE70**	EE|NOMH	24.7	No	No	No
**EE71**	EE|MH	26.5	No	No	No
**EE72**	EE|MH	26.2	No	Yes	Sí
**EE73**	EE|NOMH	25.8	No	No	Yes
**EE74**	EE|MH	26.5	No	No	No
**EE75**	EE|MH	29.0	No	No	No
**EE76**	EE|NOMH	33.0	No	No	No
**EE77**	EE|NOMH	30.4	No	No	No
**EE78**	EE|NOMH	30.7	No	No	No
**EE79**	EE|MH	23.4	No	Yes	Yes
**EE80**	EE|NOMH	25.0	No	No	Yes
**EE81**	EE|NOMH	28.6	No	No	No
**EE82**	EE|NOMH	23.3	No	No	No
**EE83**	EE|MH	25.8	No	No	No
**EE84**	EE|NOMH	26.6	No	No	No
**EE85**	EE|NOMH	22.4	No	No	No
**EE86**	EE|NOMH	21.5	No	Yes	Yes
**EE87**	EE|NOMH	26.9	No	No	No
**EE88**	EE|NOMH	29.4	No	No	No
**EE89**	EE|MH	23.9	No	No	No
**EE90**	EE|MH	27.1	No	No	No
**EE91**	EE|MH	27.5	No	No	No
**EE92**	EE|NOMH	26.2	No	No	No
**EE93**	EE|NOMH	34.0	No	No	No
**EE94**	EE|NOMH	29.3	No	No	No
**EE95**	EE|MH	32.1	Yes	No	No
**EE97**	EE|NOMH	24.4	No	No	No
**EE98**	EE|NOMH	29.4	Yes	Yes	No
**EE99**	EE|NOMH	25.8	No	No	Yes
**EE100**	EE|NOMH	28.3	No	No	No
**EE101**	EE|NOMH	24.9	No	No	No
**EE102**	EE|MH	26.6	Yes	Yes	Yes
**EE103**	EE|NOMH	26.2	No	No	Yes

**EE104**	EE|NOMH	23.7	N/A	N/A	N/A
**EE106**	EE|NOMH	23.5	N/A	N/A	N/A
**EE107**	EE|MH	28.8	No	No	No
**EE108**	EE|MH	24.8	No	No	No
**EE110**	EE|NOMH	24.5	No	No	No
**EE111**	EE|NOMH	22.6	No	No	No
**EE112**	EE|NOMH	24.2	No	No	No
**EE113**	EE|MH	21.4	N/A	N/A	N/A
**EE114**	EE|NOMH	25.7	No	No	No
**EE115**	EE|NOMH	28.6	No	No	No
**EE116**	EE|NOMH	24.1	No	No	No
**EE117**	EE|NOMH	24.8	N/A	N/A	N/A

N/A: No information available

Supplementary Table 2. Attenuation and buffering values for each individual obtained from fecal samples.

Sample	Attenuation	Buffering
EE100	3.10	2.03
EE101	3.44	1.84
EE102	3.72	1.74
EE103	2.28	2.08
EE104	3.51	1.98
EE106	3.46	1.69
EE107	3.59	1.77
EE108	2.59	2.02
EE110	3.13	1.98
EE111	3.74	1.85
EE112	3.13	1.94
EE113	2.91	2.02
EE114	2.84	2.03
EE115	3.20	2.04
EE116	2.07	1.96
EE117	3.00	1.88
EE11	3.48	1.91
EE12	3.09	2.03
EE13	2.67	1.92
EE14	2.81	2.02
EE15	3.64	1.96
EE16	3.39	1.99
EE17	3.15	1.98
EE18	3.40	1.98
EE19	3.53	1.75
EE20	3.19	1.99
EE21	2.87	2.04
EE22	2.79	1.96
EE23	2.86	2.04
EE24	2.99	1.96
EE25	2.48	2.02
EE26	3.52	1.90
EE27	2.64	2.01
EE28	2.89	2.13
EE30	2.62	1.85
EE31	3.83	1.85
EE32	2.78	1.93
EE33	2.95	2.02
EE34	2.65	2.04
EE35	3.35	1.94
EE36	3.28	1.97
EE37	3.40	1.98
EE38	3.28	2.01
EE39	2.64	2.00
EE40	2.18	2.08
EE41	2.89	2.09
EE42	3.03	2.00
EE43	2.78	1.99
EE44	2.57	1.98

EE45	2.85	2.04
EE46	3.25	1.93
EE47	2.33	2.04
EE48	3.13	2.05
EE49	3.44	2.04
EE50	3.33	1.93
EE51	1.76	2.13
EE52	2.61	2.12
EE53	2.95	2.00
EE54	2.42	2.08
EE55	3.18	2.01
EE56	2.46	2.10
EE57	3.44	1.87
EE58	2.89	1.97
EE59	2.80	1.98
EE61	2.70	1.94
EE62	2.02	2.10
EE64	2.86	2.02
EE65	3.13	1.83
EE66	1.83	1.99
EE67	3.42	1.96
EE68	2.60	1.86
EE69	3.18	2.02
EE70	1.89	2.05
EE71	2.98	2.04
EE72	3.47	1.98
EE73	2.86	2.04
EE74	2.86	2.10
EE75	3.25	1.95
EE76	3.17	1.83
EE77	2.99	1.80
EE78	3.24	1.99
EE79	2.13	1.99
EE80	3.74	1.91
EE81	3.90	1.80
EE82	2.90	2.06
EE83	2.77	2.07
EE84	3.67	1.92
EE85	3.51	1.98
EE86	3.68	1.95
EE87	3.42	1.92
EE88	2.96	2.12
EE89	2.77	2.07
EE90	2.93	2.04
EE91	1.47	2.04
EE92	3.14	1.94
EE93	3.56	1.98
EE94	3.58	1.87
EE95	3.53	1.85
EE97	3.75	1.87
EE98	3.16	1.97
EE99	3.17	2.04

Supplementary Table 3. Attenuation and buffering values for each individual obtained from saliva samples.

Sample	Attenuation	Buffering
EE100	2.48	2.05
EE101	1.95	2.10
EE102	2.83	2.00
EE103	2.39	2.08
EE104	2.36	2.06
EE106	2.64	1.98
EE107	3.14	1.93
EE108	2.42	2.05
EE110	2.63	2.04
EE111	3.00	2.02
EE112	2.99	2.03
EE113	2.66	2.04
EE114	2.95	2.02
EE115	0.84	2.33
EE116	2.63	1.98
EE117	2.50	2.04
EE11	2.84	2.02
EE12	2.76	2.01
EE13	2.58	2.03
EE14	2.28	1.92
EE15	3.22	1.94
EE16	2.59	2.04
EE17	3.07	1.87
EE18	2.98	1.97
EE19	2.53	1.96
EE20	3.50	1.90
EE21	2.42	2.04
EE22	3.26	1.93
EE23	2.80	1.98
EE24	2.92	2.03
EE25	2.43	2.00
EE26	2.12	2.14
EE27	2.27	2.01
EE28	2.45	2.09
EE30	2.77	2.01
EE31	2.77	2.06
EE32	2.42	2.10
EE33	2.32	2.07
EE34	2.51	2.04
EE35	2.70	2.05
EE36	2.66	2.03
EE37	2.73	2.02
EE38	1.35	2.09
EE39	2.63	1.97
EE40	1.26	2.11
EE41	2.84	2.04
EE42	2.78	2.02
EE43	2.10	2.13
EE44	2.59	2.03

EE45	2.94	1.98
EE46	2.61	2.07
EE47	2.60	2.08
EE48	2.67	2.04
EE49	2.75	2.07
EE50	2.46	2.06
EE51	2.84	2.02
EE52	2.45	2.06
EE53	2.88	2.02
EE54	2.85	2.03
EE55	1.75	2.03
EE56	2.99	1.78
EE57	2.61	2.04
EE58	2.63	2.06
EE59	3.00	1.98
EE61	2.69	2.02
EE62	2.84	1.99
EE64	3.01	2.02
EE65	2.14	2.00
EE66	3.12	1.96
EE67	3.20	1.91
EE68	2.80	1.99
EE69	3.17	2.00
EE70	3.00	2.01
EE71	3.16	1.97
EE72	2.85	1.98
EE73	2.62	2.05
EE74	3.01	1.96
EE75	2.92	2.02
EE76	2.69	2.02
EE77	2.64	2.05
EE78	2.62	2.06
EE79	2.49	2.01
EE80	2.69	2.03
EE81	2.84	2.04
EE82	3.05	2.00
EE83	2.54	2.05
EE84	2.85	1.93
EE85	3.09	2.01
EE86	2.73	2.05
EE87	2.74	2.01
EE88	1.42	1.94
EE89	3.13	1.95
EE90	2.62	2.02
EE91	2.41	1.90
EE92	2.65	2.06
EE93	2.29	2.06
EE94	2.41	2.09
EE95	2.81	2.01
EE97	2.54	2.06
EE98	3.03	1.96
EE99	1.68	2.06

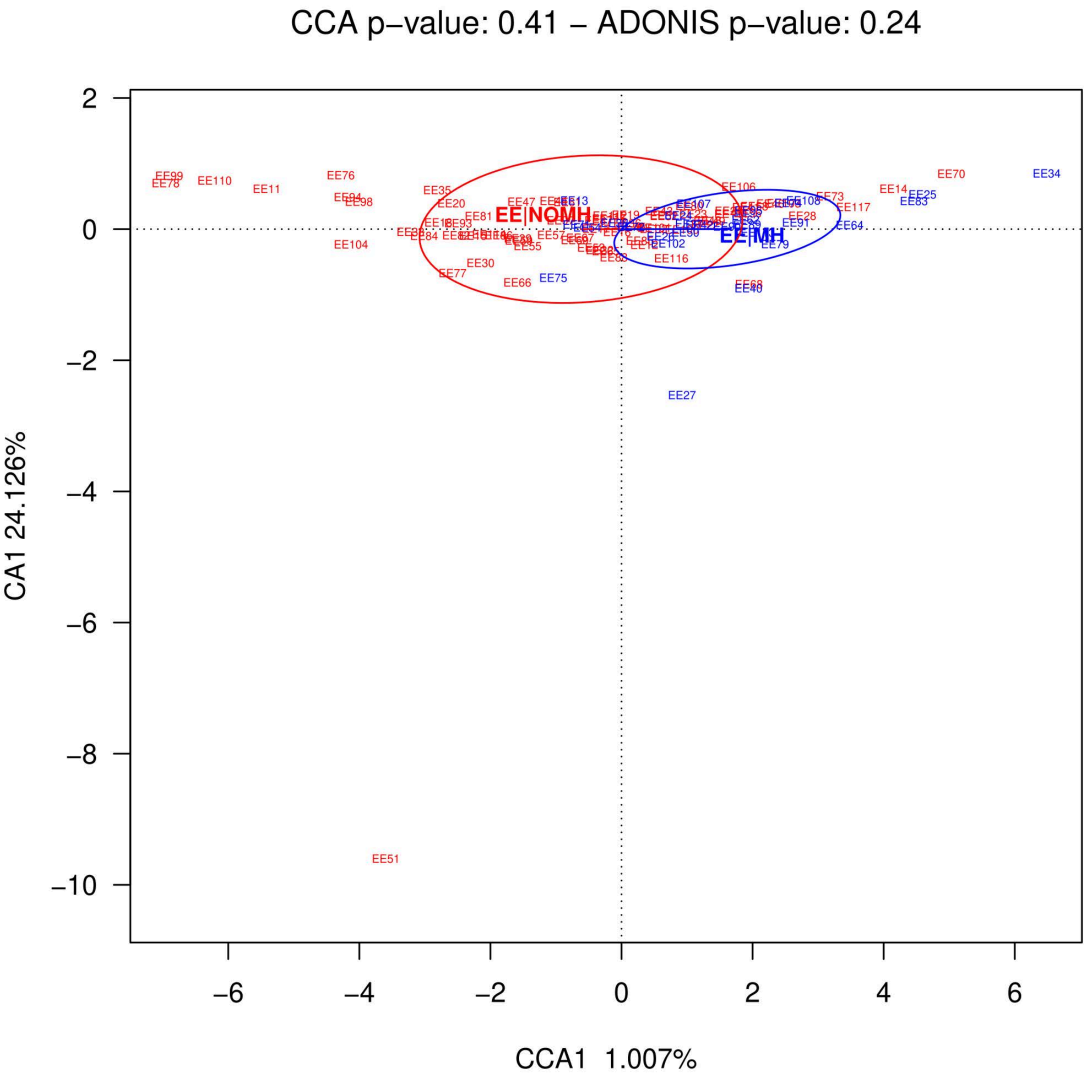

Supplementary Figure 1. Canonical Correspondence Analysis (CCA) of Functional Orthologs (KO) from metagenomic data of EE/MH (blue) and EE/NOMH (red) at the genus level.

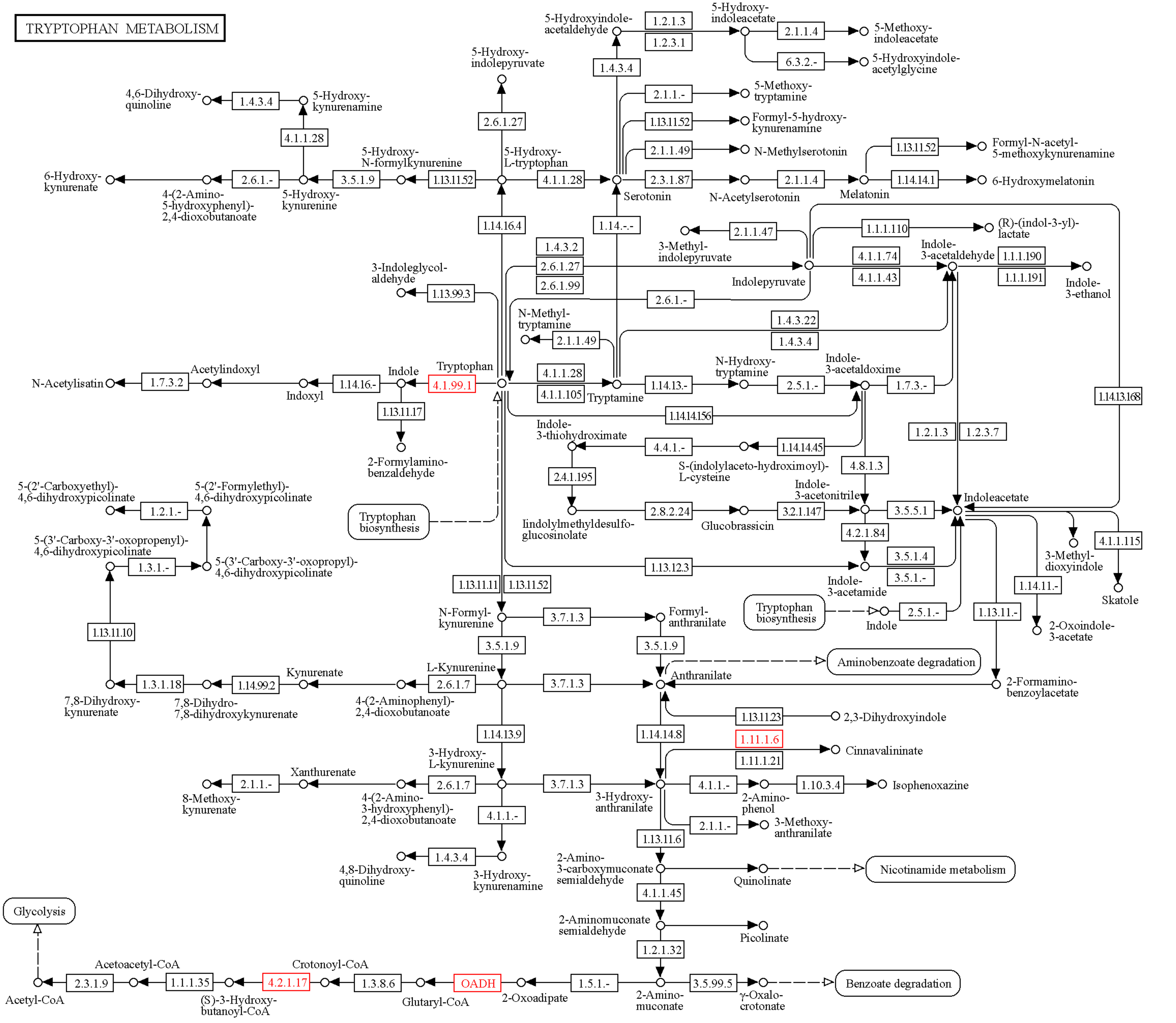

Supplementary Figure 2. Tryptophan metabolism pathway. In red, significantly different KO categories between EE/NOMH and EE/MH groups. Taken from: https://www.genome.jp/kegg/

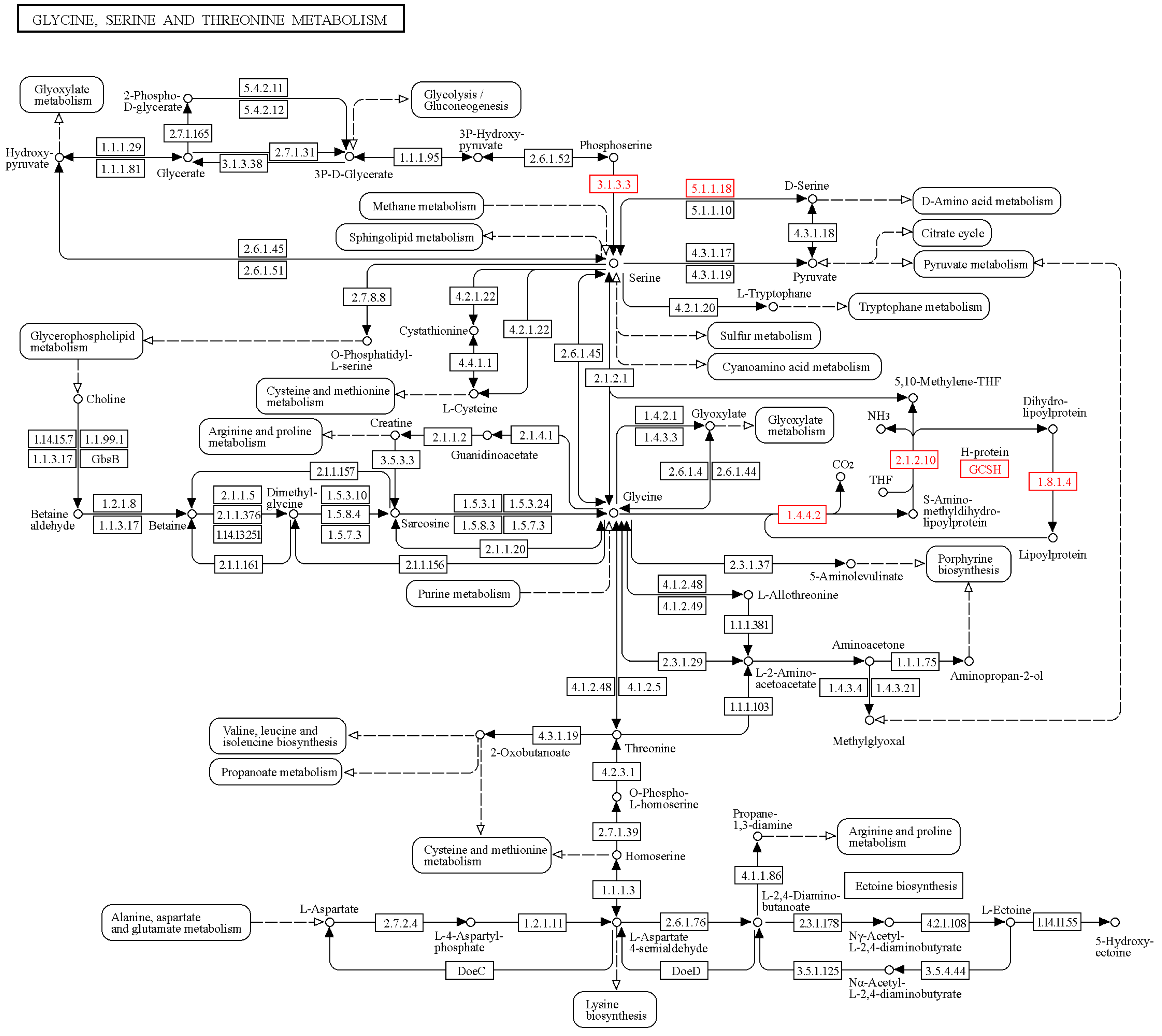

Supplementary Figure 3. Glycine, Serine, and Threonine metabolism pathway. In red significantly different KO categories between EE/NOMH and EE/MH groups. Taken from: https://www.genome.jp/kegg

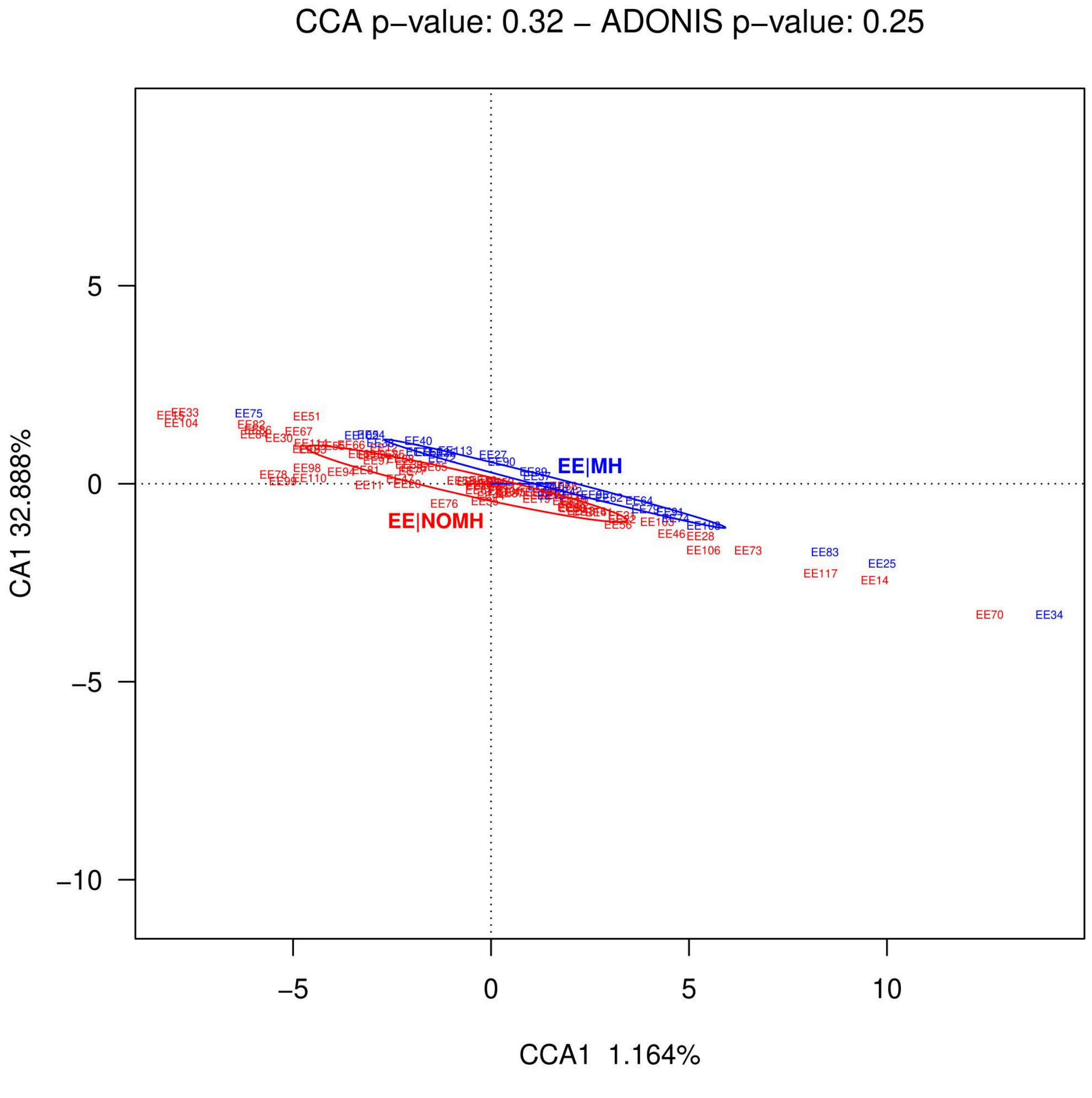

Supplementary Figure 4. Canonical Correspondence Analysis (CCA) of Kegg metabolic pathways of EE/MH (blue) and EE/NOMH (red) groups.

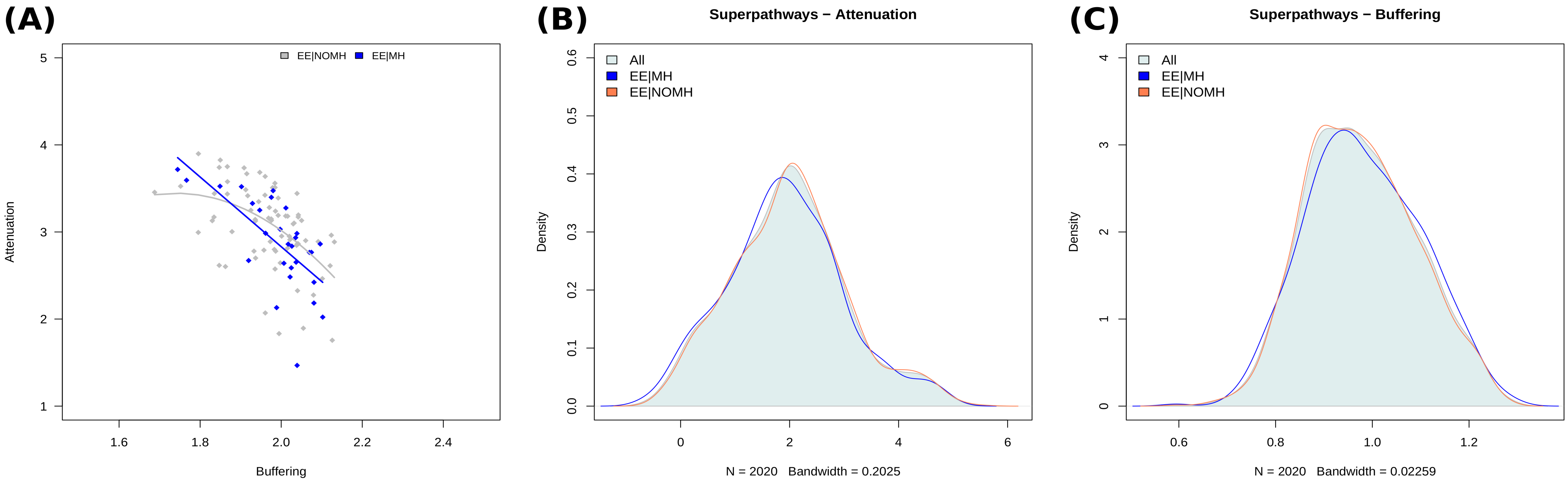

Supplementary Figure 5. (A) Values for attenuation and buffering for fecal samples from EE/MH and EE/NOMH, with lines or curves caluculated using polynomial regression. Distribution for (B) attenuation and (C) buffering considering all fecal samples and superpathways.

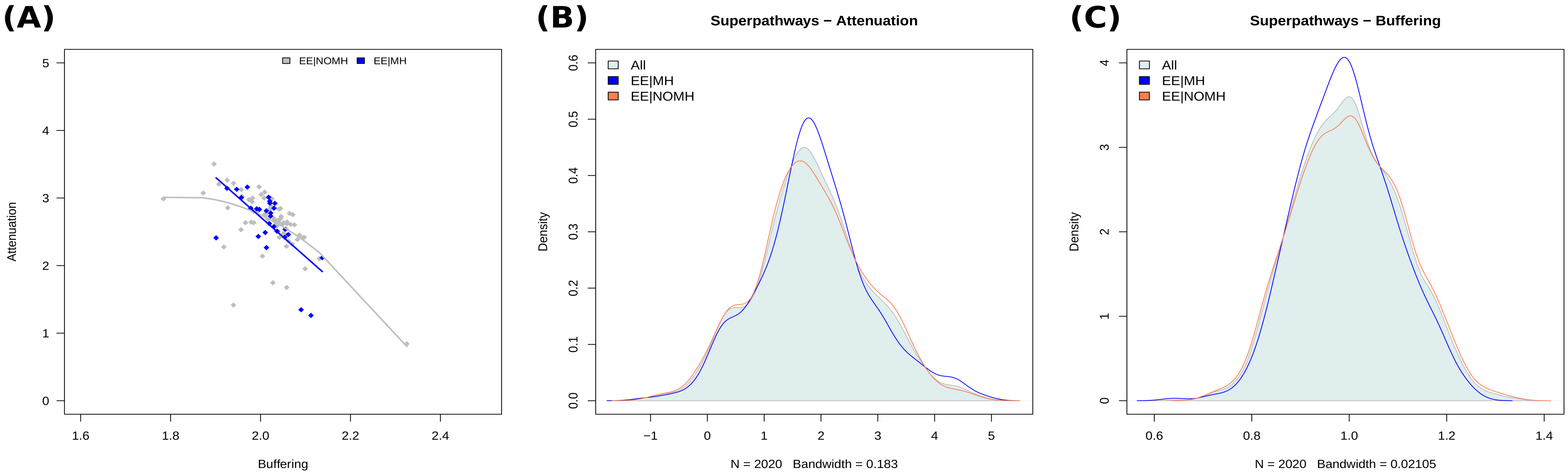

Supplementary Figure 6. (A) Values for attenuation and buffering for saliva samples from EE/MH and EE/NOMH, with lines or curves caluculated using polynomial regression. Distribution for (B) attenuation and (C) buffering considering all saliva samples and superpathways.

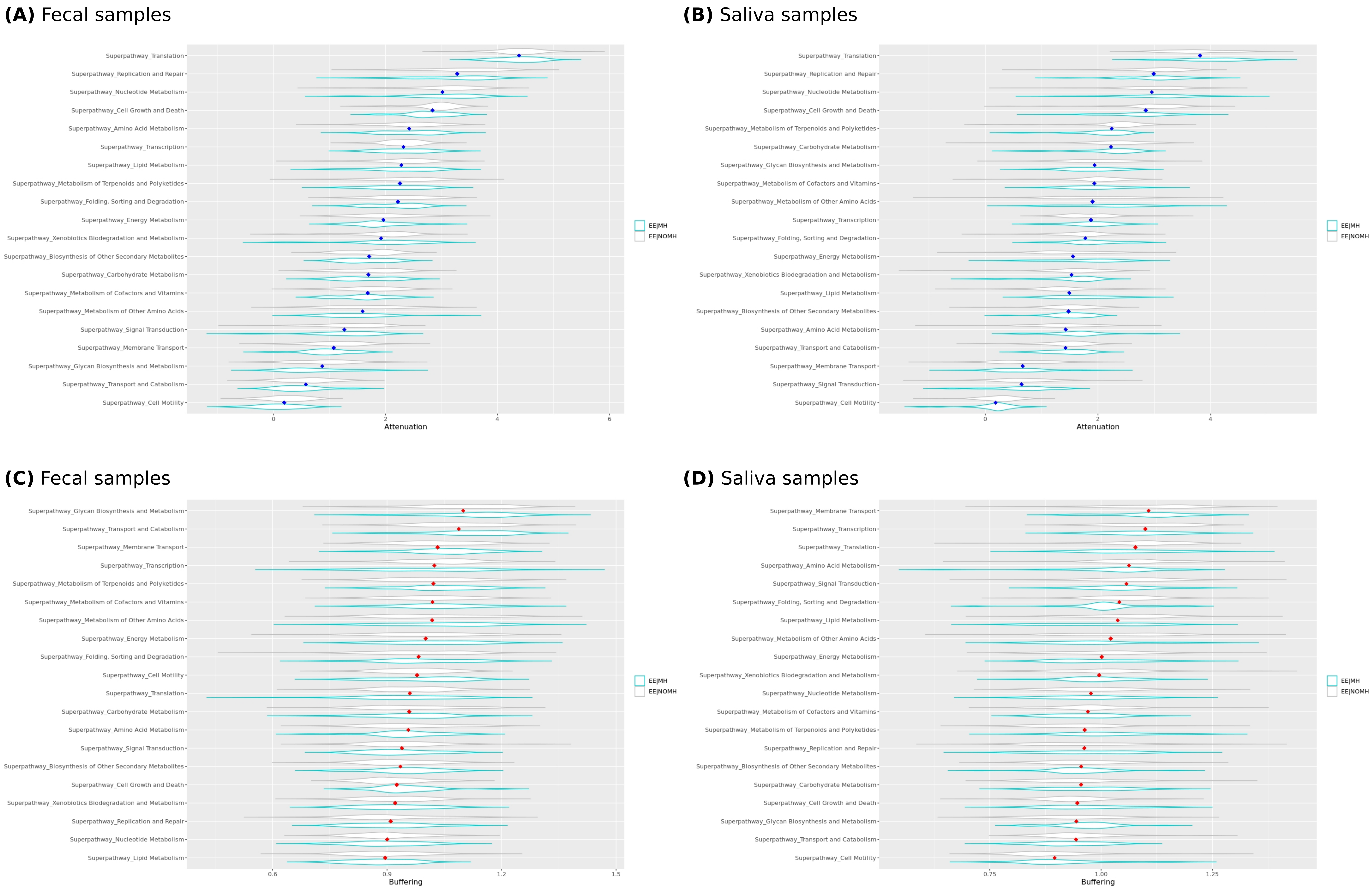

Supplementary Figure 7. Distribution of attenuation in (A) fecal samples and (B) saliva samples, and buffering in (C) fecal samples and (D) saliva samples by superpathway, for EE/MH and EE/NOMH.
